# Unified Framework for Molecular Response Functions of Different Electronic-Structure Models

**DOI:** 10.1021/acs.jpca.4c07789

**Published:** 2025-04-16

**Authors:** Bin Gao, Magnus Ringholm

**Affiliations:** Hylleraas Centre for Quantum Molecular Sciences, Department of Chemistry, UiT The Arctic University of Norway, N-9037 Tromsø, Norway

## Abstract

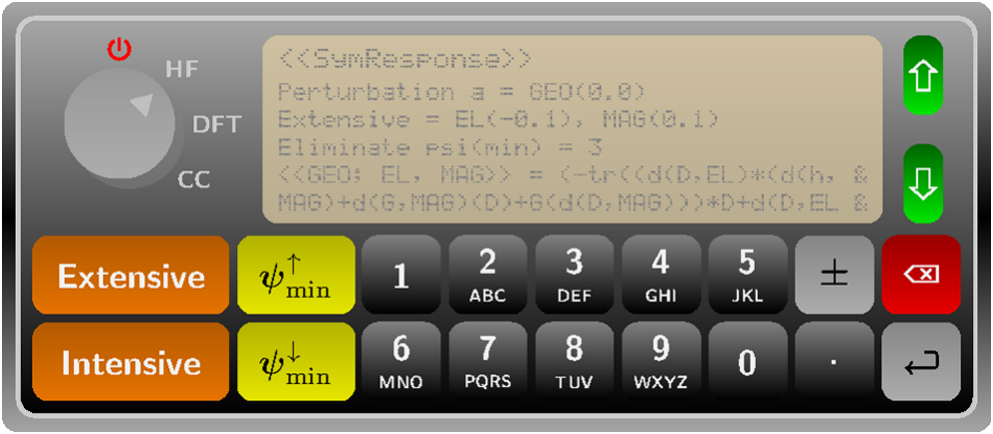

A unified framework—SymResponse—has been developed as a versatile tool to aid the implementation of response theory for different electronic-structure models. The framework manipulates the quasi-energy formulation of response theory at a symbolic level by building on top of other well-developed symbolic libraries. Response functions can therefore be nicely represented by “symbolic expressions,” which can be further evaluated numerically by users by developing their own evaluation routines with the assistance of SymResponse. The design of SymResponse makes it extensible to different electronic-structure models with only a moderate further amount of development effort. Response theory at Hartree–Fock, density functional theory, and coupled-cluster levels has been implemented in the present work as a demonstration, where elimination rules [KristensenK.J. Chem. Phys.2008, 129, 214103]19063540 10.1063/1.3023123 can also be applied to offer the possibility to reduce the size of computational tasks.

## 1 Introduction

The calculation of molecular properties has been and will stay an active research field in computational chemistry, as such calculations work as a bridge between theory and experiment. From the side of theoretical studies, the simulation of molecular properties involves solving the time-dependent Schrödinger equation under external (and usually time-dependent) influences. If these influences or *perturbations* are sufficiently weak, this can be carried out by perturbation-theory approaches. Such an approach in the framework of the Hartree–Fock approximation can be dated back to 1964 by Ball and McLachlan.^1,2^

After the seminal work by Runge and Gross^3^ in 1984, many efforts have been devoted to obtaining information about electronically excited states within the framework of time-dependent density functional theory (TDDFT). One important such contribution is by Casida in 1990s,^4,5^ in which the information on excited states is derived as a linear response to an applied perturbation. Over the last few decades, impressive achievements have been made in the use of response theory to calculate molecular properties. Such developments are mostly based on the pioneering work of Olsen and Jørgensen^6^ in 1985, in which they for the first time devised a general way of calculating higher-order response functions for self-consistent field (SCF) and multiconfigurational SCF (MCSCF) noneigenstates of the unperturbed molecular Hamiltonian. Later on, the quasi-energy approach was developed,^7−9^ in which frequency-dependent response functions could be conveniently derived from the derivatives of the time-averaged quasi-energy with respect to external perturbations. The so-called 2*n* + 1 and 2*n* + 2 rules could be routinely used for a properly chosen Lagrangian to judiciously eliminate the need to calculate some response parameters during calculations^8,10^ and thereby offering an opportunity to reduce the computational task size.

So far, the quasi-energy approach has been successfully applied to different electronic-structure models, for instance, Hartree–Fock (HF) and DFT, MCSCF, configuration interaction (CI) and coupled-cluster (CC) theory.^8,11,12^ Relatively recently, Thorvaldsen and co-workers proposed an atomic orbital (AO) density matrix-based quasi-energy formulation of the response theory at the HF and DFT levels.^13^ By its open-ended formulation, their approach can be employed to calculate a wide range of molecular properties with time- and perturbation-dependent basis sets and relativistic or nonrelativistic Hamiltonians.^14,15^ In a more recent work, Ringholm, Jonsson and Ruud presented a new implementation of this open-ended response theory formulation by using the technique of recursive programming.^16^ Their implementation has made it possible to manage the calculation of a large selection of response properties of molecular systems in an analytic manner, limited by the generality of modules for, e.g., differentiated one- and two-electron integral contributions to which this implementation is connected. An implementation of the recursive approach to the calculations of single residues of response functions has also been reported by Friese, Ringholm, Ruud and their co-workers.^17,18^

It is undoubtedly attractive to extend such open-ended recursive capability to other electronic-structure models, such as the MCSCF, CI and CC levels of theory, which are not supported in the aforementioned formulation.^13^ This can be considered in light of the observation that standard grids and aspects of the functionals themselves may be problematic when it comes to the integration of exchange-correlation (XC) functionals and their derivatives as in the case of higher-order polarizabilities^15,19^ and properties related to vibrational spectroscopies.^20^ Furthermore, and perhaps more simply, this can also be motivated by the desire to calculate analytically a wide variety of molecular properties at accuracies unattainable by the HF and DFT approaches.

However, algorithms and software development of the aforementioned recursive approach^16−18^ are specially designed for the AO density matrix and hardly reusable for other electronic-structure models. We therefore in this paper propose a new unified framework called SymResponse,^21^ which is a tool for the simulation of molecular response functions and is extensible to different electronic-structure models, offering significant reusability. The key to extensibility and reusability is an explicit separation between symbolic computations and actual evaluation in the quasi-energy formulation of response theory. The separation is achieved by building on earlier work by Gao^22,23^ where key operators and function(al)s are programmed to different C++ symbolic classes, so that response functions can be computed straightforwardly from symbolic arithmetic and differentiation. SymResponse is designed to generate hierarchical symbolic representations of the contributions to the terms one would need to evaluate for a desired property at a given level of theory. It will provide such a representation at a significant “depth” or level of detail, and while functionality for the actual (numerical) evaluation of the contributions identified by SymResponse must be supplied externally, SymResponse nevertheless offers the opportunity to significantly ease the development and maintenance effort in this area.

As a demonstration, we will in this work present the implementation of AO density matrix-based^13^ and coupled-cluster^8^ response theories in SymResponse. To illustrate the functionality, we will apply it to the evaluation of response functions of a two-level atom model within the AO density matrix-based response theory, where response functions can be evaluated analytically.^24^ Our results highlight the capabilities of SymResponse to automate the obtainment of response function expressions as these expressions become increasingly complicated at higher orders of perturbation.

The rest of this paper is organized as follows: First, we will briefly describe the theoretical background applied in the current work—the quasi-energy response theory in AO density matrix-based^13^ and coupled-cluster^8,11,25,26^ formulation. Next, we will present the design and implementation of SymResponse, and special consideration will be given to good code reusability when extending to include more electronic-structure models. Then, we will demonstrate the functionality of SymResponse by applying it to the computations of response functions within the AO density matrix-based and coupled-cluster response theories, in particular the two-level atom model will be investigated in the case of the AO density matrix-based formulation. Finally, we will give our concluding remarks and future work.

## 2 Theoretical Background

We will in this section consider basic expressions of the AO density matrix-based and coupled-cluster response theories. We here choose the quasi-energy derivative formulation and start from a phase-isolated state |Φ(*t*)⟩^13^
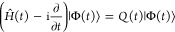
1where the Hamiltonian *Ĥ*(*t*) is expressed as
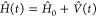
2where *Ĥ*_0_ is a time-independent unperturbed Hamiltonian, and *V̂*(*t*) is a time-dependent operator for some applied perturbation or perturbations. The quantity *Q*(*t*) is the time-dependent quasi-energy
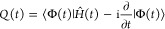
3and is a real-valued function when the Hamiltonian *Ĥ*(*t*) is Hermitian.^8,13^

It is *Q*(*t*) or the time-dependent quasi-energy Lagrangian^8,12^
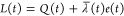
4which forms the starting point for approaches for the obtainment of molecular properties described in this work. Here *e*(*t*) represents constraints that the system has to obey, such as the time-dependent self-consistent-field (TDSCF) equation and the idempotency constraint in the AO density matrix-based response theory, or the amplitude equations in the coupled-cluster response theory. λ̅(*t*) contains the associated time-dependent Lagrangian multipliers.

A fundamental aspect of the quasi-energy derivative method is that we require *V̂*(*t*) to be periodic with a period *T*, and we express it on a general form^15^
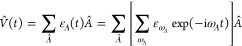
5where *ε*_*A*_(*t*) is a real-valued time-periodic function representing the perturbation strength of the time-independent one-electron operator *Â*. To ensure the hermiticity of *V̂*(*t*), the operator *Â* should be Hermitian.

The sum in the brackets [···] of eq 5 is the Fourier series of *ε*_*A*_(*t*). In order for *ε*_*A*_(*t*) to be real-valued, its Fourier series should contain both frequencies ω_*A*_ and −ω_*A*_, and the complex Fourier coefficients *ε*_ω_*A*__ should satisfy *ε*_–_ω_*A*_ = *ε*_ω_A__^*^. Due to the assumed periodicity of *V̂*(*t*), each frequency ω_*A*_ can be expressed as an integer *z*_*A*_ times some fundamental frequency ω_*A*_ = *z*_*A*_ω_0_. Still because of the periodicity of *V̂*(*t*), the Hamiltonian *Ĥ* and the state |Φ(*t*)⟩ are also time-periodic with the same period *T*. One can therefore consider the time-averaged quasi-energy Lagrangian
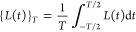
6which fulfills the variational condition
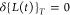
7

The molecular response functions can be related to the time-averaged quasi-energy Lagrangian as^8,12^
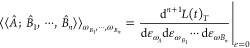
8where
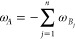
9and where {*ε*} = 0 denotes the evaluation at zero perturbation strength, i.e., *ε*_ω_*A*__, *ε*_*ωB*_1__, ···, *ε*_*ωB*_*n*__ = 0.

To evaluate the response functions in eq 8, a typical procedure is to first identify all required quantities involved in this equation, and then to compute these quantities from the equation of motion as deduced from the variational condition, eq 7.^8^

In the following subsections, we will first introduce the necessary definitions and notations about perturbations and derivatives for the current work. Next, we will describe key formulas of the response functions and response equations in the AO density matrix-based and coupled-cluster formulation, as well as the use of elimination rules to reduce the number of response parameters to be solved.^10,13,16^

### 2.1 Perturbations and Derivatives

We will in this section establish some concepts related to collections of perturbations that will be useful in the derivation to come. Here, a perturbation *a* can be associated with characteristics such as its perturbation strength *ε*_ω_*A*__, the associated operator *Â*, or the frequency ω_*A*_. Two perturbations are equivalent or the same if and only if they have the same characteristics.

Throughout the present work, we use “perturbation multichain” to represent derivatives with respect to perturbation strength. We define a perturbation *multichain*(27) as *a totally ordered multiset* of perturbations *a* ≤ *b* ≤ *c* ≤ ···, and is denoted as *abc*··· for the sake of simplicity. We note that the specific way in which the binary relation “≤” is defined is arbitrary for the purposes of this presentation.

Derivatives with respect to perturbation strength can thus be expressed in a more compact way by using the perturbation multichain notation. For example, derivatives of the time-averaged quasi-energy Lagrangian (8) can be written as . We notice that the notion of a perturbation collection/tuple or a totally ordered tuple of perturbation strengths have also been proposed and used in expressing these derivatives.^13,22,28^ Our perturbation multichain can be viewed as a “totally ordered” tuple^27^ of perturbations with respect to which derivation of response-theory expressions can be straightforwardly performed.

As with the totally ordered tuple of perturbation strengths,^22^ the following properties hold for the perturbation multichain:

1. The length of a perturbation multichain is the order of its corresponding derivative;
2. Perturbations that are equivalent according to the binary relation “≤” must always be consecutive in the multichain. For example, *abbc* can be a valid perturbation multichain, but *abcb* is not.
3. Multichains *abc* ≠ *acb*, because *b* ≤ *c* in the former case while *c* ≤ *b* in the latter case.
  These two multichains may represent derivatives stored in tensors with different shapes. For example, let *a*, *b* and *c* be the electric dipole, magnetic dipole and geometrical perturbations. The shapes of derivative tensors are (3, 3, 3*N*) and (3, 3*N*, 3) respectively for multichains *abc* and *acb*, where *N* is the number of atoms.

To represent derivatives of a product in response theory in a more practical manner, we will also use the concept “perturbation index chain” and its “set partition”. A perturbation index chain^22^ is a totally ordered set (or a chain) {1, ···, *n*} whose elements are unique and each points at the position of its corresponding perturbation in a perturbation multichain *b*_1_ ··· *b*_*n*_, or with a more short-hand notation, *b*_{1, ···, *n*}_. The set partition^22,29^ of a perturbation index chain {1, ···, *n*} is a collection of *k* (1 ≤ *k* ≤ *n*) nonempty and disjoint subchains (or “blocks”) of {1, ···, *n*}.

For example, the *n*th-order derivative of the second term in eq 4 can, using the concepts introduced above, be written as
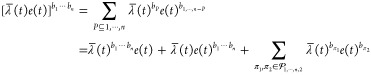
10where *P* is a subchain of the perturbation index chain {1, ···, *n*} and  represents the set of all set partitions of the chain {1, ···, *n*} with exactly *k* (1 ≤ *k* ≤ *n*) blocks π_1_, ···, π_*k*_.

In practice, high-order derivatives of a product are obtained by calling a function differentiate (see Table 1) in our previously developed library Tinned,^23^ which generates a representation of such derivatives in an order-by-order manner. More details about the implementation of symbolic differentiation can be referred to ref (22).

**Table 1:** New Contributions to Tinned^23^ for the Development of AO Density Matrix-Based and Coupled-Cluster Response Functionality

classes and helper functions	description
class PertMultichain	A C++ std::multiset container representing a perturbation multichain.
class PerturbedParameter(name)	A (response) parameter with a given name. The parameter will depend on all perturbations to arbitrary order. It can be used to represent, for example, Lagrangian multipliers.
clean_temporum(x)	Remove the following quantities from x: (i) unperturbed time-differentiated quantities and unperturbed **T̃** matrix [eq 18 in ref (22)], and (ii) their perturbed ones but with zero sum of frequencies, see for example, eq 32.
differentiate(expr, perturbations)	differentiate expr with respect to given perturbations one by one.
eliminate(x, parameter, perturbations, min_order)	eliminate a given response parameter’s derivatives from x. maximum order of derivatives to be eliminated is the length of perturbations, and minimum order is specified by min_order.
latexify(x)	write x in LATEX.
make_conjugate_transpose(arg)	make a conjugate transpose of arg that can be used, for example, to construct the coupled-cluster time-averaged quasi-energy Lagrangian (20).
replace_all<T>(x, subs_dict)	replace some symbols with others (specified by the parameter subs_dict) in x. These symbols have the same type T and their derivatives will also be replaced with corresponding ones.

### 2.2 AO Density Matrix-Based Response Theory

We will in this section recapitulate the AO density matrix-based quasi-energy formulation proposed by Thorvaldsen et al.,^13^ which together with the coupled-cluster approach introduced in the next section are the two levels of theory at which SymResponse is utilized in this work. The key quantity in this formulation is the so-called variational time-averaged quasi-energy derivative Lagrangian,^13^
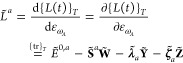
11where the notation  denotes a trace over the resulting matrix-shape expression on the right-hand side followed by an average over a time period *T* of the applied perturbations. The tildes above symbols are used to denote quantities considered at general perturbation strengths {ε}, instead of (or not yet evaluated at) zero perturbation strength.

The right-hand side terms of (11) are,^13,16,22^ respectively, the generalized energy *Ẽ* (where the first superscript denotes the order of differentiation of *Ẽ* with respect to the AO density matrix **D̃**), the overlap matrix **S̃**, the generalized energy-weighted density matrix **S̃**, and the TDSCF equation **Ỹ** and idempotency constraints **Z̃**, with respective Lagrangian multipliers **λ̃**_*a*_ and **ζ̃**_*a*_.

The response functions can be related to the variational time-averaged quasi-energy derivative Lagrangian (11) as^13^
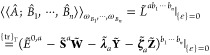
12which follows the *n* + 1 formulation,^10,13^ i.e., molecular properties to order *n* + 1 may be determined by response parameters (here are perturbed AO density matrices) to no greater than order *n*. The response functions can be further simplified by reducing the number of perturbed AO density matrices and Lagrangian multipliers according to the elimination rules developed in ref (10). We will discuss these rules in a later subsection together with similar considerations for coupled-cluster response theory.

To evaluate the response functions (12), one must determine derivatives of the AO density matrix in the frequency domain. The *n*th-order such derivatives can be partitioned as^13^
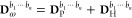
13where the *particular* solution **D**_P_^*b*_1_···*b*_*n*_^ and the *homogeneous* solution **D**_H_^*b*_1_···*b*_*n*_^ are represented as^13^
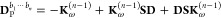
14
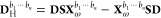
15The term **K**_ω_^(*n*–1)^ is obtained by truncating the derivatives of the AO density matrix after (*n* – 1)-th order in the *n*th-order derivatives of the idempotency constraint **Z̃** in the frequency domain,^13^ that is,
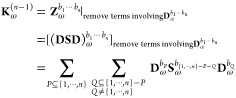
16The *n*th-order response parameter **X**_ω_^*b*_1_···*b*_*n*_^ is found by solving the linear response equation^13^
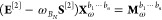
17where **E**^[2]^ and **S**^[2]^ are respectively the generalized Hessian and metric operator,^13^ and
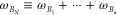
18The right-hand side **M**_ω_^*b*_1_···*b*_*n*_^ is obtained from the *n*th-order derivative of the TDSCF equation **Ỹ** in frequency domain and by replacing the *n*th-order derivative of the AO density matrix **D**_ω_^*b*_1_···*b*_*n*_^ with its corresponding particular solution D_P_^*b*_1_···*b*_*n*_^, i.e.^16^
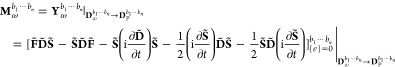
19where **F̃** is the generalized AO Fock matrix.^13,22^

### 2.3 Coupled-Cluster Response Theory

We now turn to an exposition of coupled-cluster response theory in a similar vein to the AO density matrix-based formulation in the previous subsection. Neglecting orbital relaxation^11,30^ in the present work, the time-dependent coupled-cluster quasi-energy Lagrangian is^8,11,25,26^
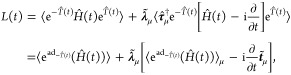
20where we have used the Einstein summation convention for μ. Notations ⟨*Ã*⟩ and ⟨*Â*⟩_μ_ represent expectation values of an operator *Â*, and are defined as
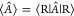
21
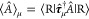
22where |R⟩ is the (Hartree–Fock) reference state. The Hamiltonian *Ĥ*(*t*) is defined in eq 2, and the time-dependent cluster operator *T̂*(*t*) is
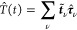
23where the row vectors ***t*~** and **τ̂** contain respectively time-dependent coupled-cluster amplitudes and (commuting) excitation operators. The row vector **λ̃** is the associated Lagrangian multipliers of the time-dependent coupled-cluster amplitude equations.^11^

The operator e^ad_–*T̂*(*t*)_^(*Ĥ*(*t*))≡ e^–*T̂*(*t*)^*Ĥ*(*t*)e^*T̂*(*t*)^ is the similarity-transformed Hamiltonian,^11^ or, using a more compact name, “exponential map,”^22^ which can be expanded as
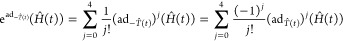
24by noticing that the Hamiltonian *Ĥ*(*t*) has no more than two-body interactions so that the expansion can be terminated after 4-fold commutators.^31^ The notation (ad_*T̂*(*t*)_)^*j*^ (*Ĥ*(*t*)) is an “adjoint map”(or adjoint representation) from Lie algebra^32^ and is defined as
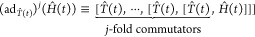
25The use of exponential and adjoint maps from Lie algebra can facilitate the development and implementation of symbolic computations and differentiation for coupled-cluster response theory as illustrated in ref (22).

The key quantities for evaluating coupled-cluster response functions are the derivatives of the coupled-cluster amplitudes ***t***_ω*B*_1_···ω*B_n_*_^*b*_1_···*b_n_*^ and the Lagrangian multipliers **λ**_ω*B*_1_···ω*B_n_*_^*b*_1_···*b_n_*^ the frequency domain. As presented in the Supporting Informationcc_response_formulation.pdf, the response equations for the *n*th-order derivatives ***t***_ω*B*_1_···ω*B_n_*_^*b*_1_···*b_n_*^ and λ_ω*B*_1_···ω*B_n_*_^*b*_1_···*b_n_*^ read
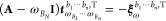
26
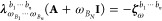
27where the superscript “*T*” denotes the transposition of row vectors, the sum of frequencies ω_*B*_*N*__ is defined in eq 18, **I** is an *n* × *n* identity matrix and **A** is the nonsymmetric coupled-cluster Jacobian.^11,25^ The right-hand side vectors **ξ**_ω_^*b*_1_···*b*_*n*_^ and **ζ**_ω_^*b*_1_···*b*_*n*_^ can be written compactly as
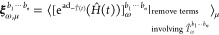
28
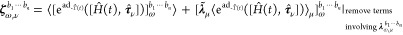
29

In the *n* + 1 formulation, the response functions can be expressed as
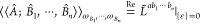
30where  indicates we take the real part of the derivative of the quasi-energy Lagrangian,^25^ and  can be determined from
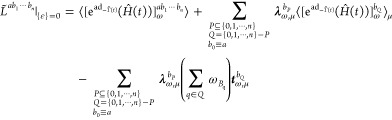
31by using the product rule, subperturbation multichains, and
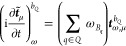
32

In the next subsection, we will present how to reduce the number of perturbed coupled-cluster amplitudes and Lagrangian multipliers to be solved according to the elimination rules in ref (10).

### 2.4 Elimination of Response Parameters

We will in this section show how we can apply the elimination rules in ref (10) to the context of the response-theory formulations shown above. By following ref (10), we divide perturbations into two categories: “intensive” and “extensive” perturbations, respectively. The former refers to perturbation whose number of components is independent of the system size, such as electric dipole and magnetic dipole perturbations that always have only Cartesian *x*, *y*, and *z* components. In contrast, extensive perturbations refer to perturbations whose number of components is proportional to the size of the molecule, like for example nuclear displacements in geometrical differentiation. Consequently, extensive perturbations usually have more components than intensive ones, and it is therefore of interest to apply elimination rules separately to the extensive perturbations to the greatest extent possible in order to maximally reduce the number of extensive response parameters to be solved.^10^

We first consider elimination rules for response functions in coupled-cluster response theory. Let *a*, *b*_1_, ···, *b*_*k*–1_ be extensive and *b*_*k*_, ···, *b*_*n*_ be intensive perturbations. We then have
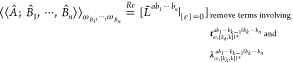
33where we have followed the notation in ref (10), that is, ***t***_ω,[*k*_***t***_,*k*]|*_^ab_1_···*b*_*k*–1_|*b*_*k*_···*b*_*n*_^ represents perturbed coupled-cluster amplitudes at orders greater than or equal to *k*_*t*_ and less than or equal to *k* when perturbations *a*, *b*_1_, ···, *b*_*k*–1_ are involved, and **λ**_ω,[*k*_**λ**_,*k*]|*_^ab_1_···*b*_*k*–1_|*b*_*k*_···*b*_*n*_^ stands for perturbed Lagrangian multipliers at orders from *k*_**λ**_ to *k* when perturbations *a*, *b*_1_, ···, *b*_*k*–1_ are involved. Orders *k*_*t*_ and *k*_**λ**_ can be chosen according to the conditions^10^
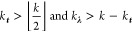
34where  is the greatest integer less than or equal to *k*/2. We note that this also includes the case *k*_*t*_ > *k*, which implies no perturbed coupled-cluster amplitudes will be eliminated and all Lagrangian multipliers can be removed.

For the AO density matrix-based response theory, it is the time-averaged quasi-energy derivative Lagrangian *L̃*^*a*^ that is variational in the multipliers **λ̃**_*a*_, **ζ̃**_*a*_ and density matrix **D̃**,^13^ and the application of elimination rules is therefore only relevant to consider for perturbations *b*_1_, ···, *b*_*n*_. Let *b*_1_, ···, *b*_*k*_ be extensive and *b*_*k*+1_, ···, *b*_*n*_ be intensive perturbations, respectively. The response functions in the AO density matrix-based response theory can be written as^10^
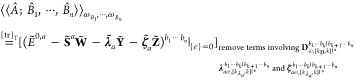
35where we can choose elimination parameters satisfying the conditions
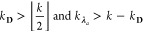
36where, similarly, *k*_**D**_ > *k* indicates no perturbed AO density matrices will be eliminated and all Lagrangian multipliers can be removed.

We note in passing that when *k* = *n* in eqs 33 and 35, i.e., when all perturbations are treated on an equal footing, one can reproduce the well-known 2*n* + 1 rule for the wave function parameters and 2*n* + 2 rule for the multipliers.^10^

## 3 Design and Implementation

In this section, we will present the design of our library SymResponse^21^ and its implementation of the AO density matrix-based^13^ and coupled-cluster^8^ response theories. Before presenting the design and implementation details, we first clarify the meaning we assign to the terms “(symbolic) computation” and “evaluation”. Unless otherwise stated, they have the following specific meaning:

**Computation or symbolic computation** is the action of calculation associated with the obtainment of a symbolic representation of a response function, and does not imply actual (numerical) evaluation;

**Evaluation** refers to the process of obtaining the actual result of the evaluation of a SymResponse symbolic representation of a response function, and would usually involve (although is not necessarily limited to) numerical actualization of the symbolically represented terms, or, in informal terms, “number-crunching”.

**Figure 1:**
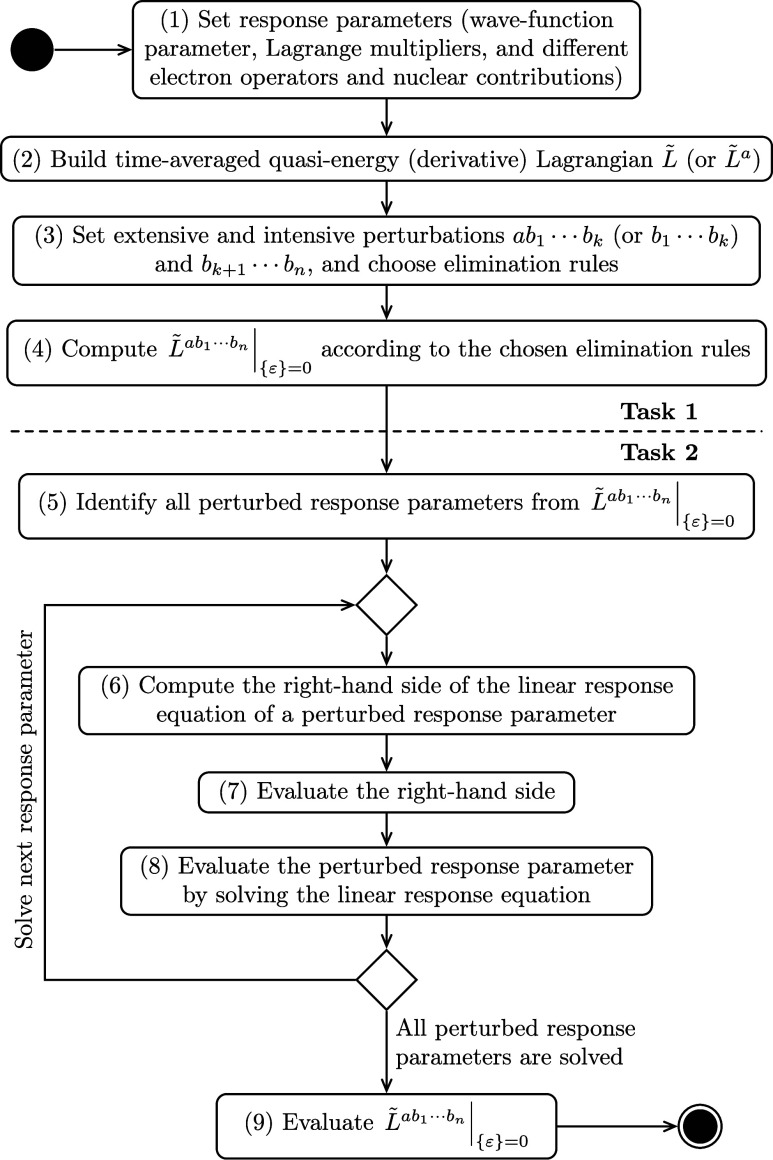
UML activity diagram of the computational structure of response theory for different electronic-structure models.

The primary objective of SymResponse is to develop a unified framework for the **symbolic computation** of molecular response functions at different electronic-structure levels, including but not limited to, HF, DFT and coupled cluster. SymResponse also aims at assisting in the **evaluation** of resulted symbolic representation of a response function, but does not include the complete fulfillment of this part in its scope. We will now examine the procedure of the application of response theory involving SymResponse. Although the particulars of this workflow may vary significantly between different electronic-structure models, we can still identify and depict overall steps as shown in Figure 1.

In this figure, we use the activity diagram of unified modeling language (UML)^33^ to represent the workflow, and we divide it into the following two overall tasks:

**Task 1 symbolic computation** consists of activities (1)–(4) that compute a response function symbolically by using, for example, eq 33 for the coupled-cluster response theory or (35) for the AO density matrix-based response theory;

**Task 2 evaluation** is divided into activities (5)–(9) that together evaluate the computed symbolic expression of the response function, in which activities (5)–(6) operate at a symbolic level and activities (7)–(9) are usually carried out involving numerical evaluation and would likely be the most time-consuming ones.

The above organization and its division into the stated activities facilitate explicit separation between symbolic computation and actual evaluation regardless of electronic-structure model. Among these Figure 1 activities, SymResponse deals with the implementation of activities (2), (4) and (6), while the remaining ones would be carried out by users with the assistance of SymResponse and two other symbolic libraries SymEngine^34^ and Tinned.^23^

SymEngine is a well-developed C++ symbolic library, and we in the present work use its modified version at https://github.com/bingao/symengine, where derivatives of symbolic matrices have been implemented. Tinned is built on SymEngine, and is furthermore geared toward response theory programming by providing classes for various electron operators and nuclear contributions, as well as helper functions for symbolic search, removal and replacement. An overview of available symbolic classes and helper functions from Tinned can be found in ref (22) and the library Tinned itself.^23^ In this manuscript, we highlight a few new classes and helper functions developed in Tinned for the purpose of the present work, as shown in Table 1. These classes and functions are intended to assist in the development effort of response theory for different electronic-structure models, and their use will be described in detail in the following subsections, where we will also describe how the above two overall tasks are approached in SymResponse, and in particular how its extensibility and reusability for different electronic-structure models can be accomplished.

### 3.1 Task 1: Symbolic Computation of Response Functions

In task 1, activities (1) and (3) would, as mentioned above, be carried out by users by using Tinned and SymEngine and can be considered as having more of a “setup” character, and we therefore do not present any particulars of their implementation here. Interested readers are referred to ref (22) and the libraries^23,34^ for more information about this topic.

**Figure 2:**
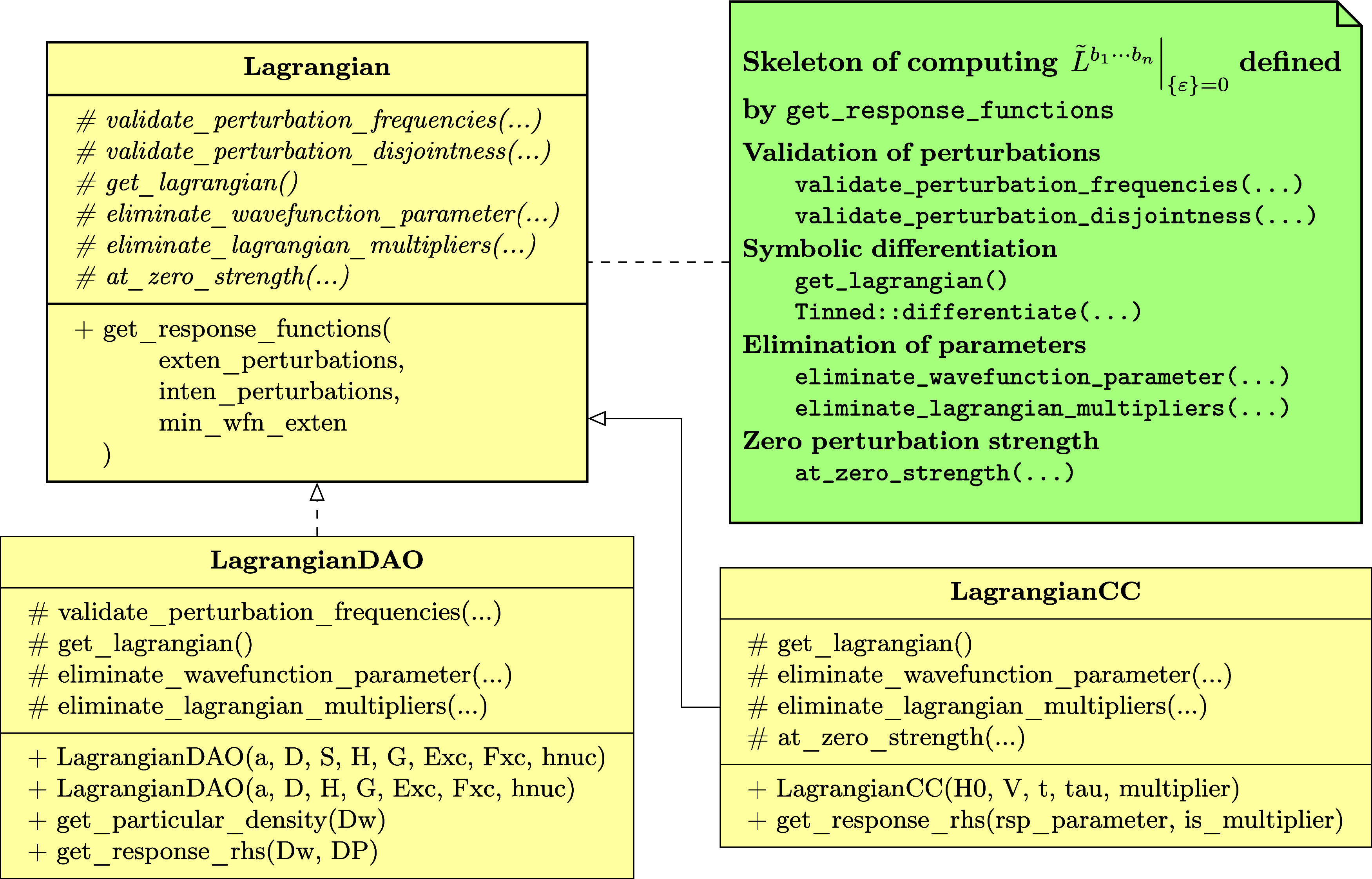
UML class diagram of SymResponse:^21^ an abstract base class Lagrangian, and two subclasses LagrangianDAO and LagrangianCC respectively for the AO density matrix-based and coupled-cluster response theories. Notations “#” and “+” respectively denote protected and public member functions of a class. Details regarding the member functions are provided in the relevant context of Section 3.

Activity (2) constructs the time-averaged quasi-energy (derivative) Lagrangian *L̃* (or *L̃*^*a*^ for the AO density matrix-based formulation), which as presented in the theoretical background, is specific to the chosen electronic-structure model. As shown in Figure 2, we have developed two classes LagrangianDAO and LagrangianCC respectively for the AO density matrix-based and coupled-cluster response theories in the current version of SymResponse. Constructors of these two classes can be straightforwardly implemented by using Tinned,^23^ and by respectively following eqs 11 and 20. Extending to include more electronic-structure models is feasible and not demanding by building on top of Tinned and SymEngine.

Input parameters for the constructors of LagrangianDAO and LagrangianCC can be easily recognized: the perturbation *a*, **D̃**, **S̃**, one-electron operators, two-electron matrix with scaled (scaling factor is γ) exchange^13^**G̃**^γ^(**D̃**), exchange-correlation energy functional *Ẽ*_xc_(ρ̃), density functional derivative matrix^13,28^**F̃**_xc_(ρ̃) and nuclear contributions for the class LagrangianDAO, and *Ĥ*_0_, *V̂*(*t*), ***t*~**, **τ̂** and **λ̃** for the class LagrangianCC. The second constructor of the class LagrangianDAO works for the case of orthonormal basis sets, where as shown in the Supporting Informationdao_response_orth.pdf, the overlap matrix **S̃** is not needed as an input parameter. Furthermore, for both constructors of the class LagrangianDAO, we use multipliers **λ̃**_*a*_ and **ζ̃**_*a*_ as symbols representing themselves with no further detail before applying the elimination rule (35), after which their representation will be expanded in terms of their constituent terms according to (un)differentiated renderings of ansätze^13^
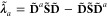
37
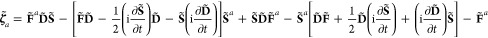
38

Next, we consider activity (4), the symbolic differentiation of *L̃* at zero perturbation strength, which can be further divided into the following four steps:

**Validation of perturbations** checks, for example, whether the sum of all perturbations’ frequencies is zero, and whether extensive and intensive perturbations are disjoint, i.e., a perturbation must not be both extensive and intensive.

One should notice that the perturbation *a* should also be considered for the AO density matrix-based response theory in this step.

**Symbolic differentiation** computes symbolic derivatives *L̃*^*ab*_1_···*b*_*n*_^ of the time-averaged quasi-energy (derivative) Lagrangian, which can be performed by simply calling the function differentiate in Tinned^23^ (see Table 1), with expr being *L̃* (or *L̃*^*a*^) from the constructor and perturbations the union of extensive and intensive perturbations.

**Elimination of parameters** eliminates wave function parameter(s) and Lagrangian multipliers from computed *L̃*^*ab*_1_···*b*_*n*_^, and which can be achieved by calling the function eliminate in Table 1 twice with parameter respectively representing wave function parameter(s) and multipliers.

The argument x is the computed *L̃*^*ab*_1_···*b*_*n*_^ or the output from the previous calling of the function eliminate. The argument perturbations contains the extensive perturbations, while min_order is the minimum order of parameter’s derivatives to be eliminated that should satisfy eq 34 or 36.

**Zero perturbation strength** mostly processes time-differentiated quantities (as well as **T̃** matrix^22^ for the AO density matrix-based response theory), as which may become zero at zero perturbation strength and should be removed from *L̃*^*ab*_1_···*b*_*n*_^, including

- unperturbed time-differentiated quantities, and
- perturbed ones but with zero frequency sum, see for example, eq 32.

These can be performed by calling the function clean_temporum in Table 1, with x being the eliminated *L̃*^*ab*_1_···*b*_*n*_^.

To achieve good code reusability and maintainability, we have chosen to employ the so-called template method pattern^35^ for the development of activity (4). In brief, this design pattern defines the skeleton of an algorithm in a base class and then defers some steps of the algorithm to subclasses. In this way, subclasses can redefine certain steps of the algorithm without changing its overall structure^35^ and this matches our design requirements for activity (4). For example, as shown in Figure 2, we have introduced an abstract base class Lagrangian, which has implemented a “template method” get_response_functions that defines the skeleton of computing  in four steps or six function calls. The subclasses LagrangianDAO and LagrangianCC must provide or can redefine certain functions of the computation without changing the overall structure of the method get_response_functions:

- Functions validate_perturbation_frequencies, validate_perturbation_disjointedness and at_zero_strength can be redefined by subclasses, respectively for the steps **Validation of perturbations** and **Zero perturbation strength**.
  For example, one needs to redefine the function validate_perturbation_frequencies for the AO density matrix-based response theory to verify that the frequency of the perturbation *a* is the negative sum of all of the other perturbations’ frequencies.
  For the coupled-cluster response theory, undifferentiated perturbation operators *V̂*(*t*) will become zero and should be removed at zero perturbation strength, which can be achieved by reimplementing the function at_zero_strength.
- Functions get_lagrangian, eliminate_wave function_parameter and eliminate_lagrangian_multipliers must be implemented by subclasses, which will be invoked by the method get_response_functions to retrieve the time-averaged quasi-energy (derivative) Lagrangian for the step **Symbolic differentiation**, and to perform the step **Elimination of parameters**, respectively.
  As aforementioned, the subclass LagrangianDAO must replace (un)perturbed Lagrangian multipliers **λ̃**_*a*_ and **ζ̃**_*a*_ by their corresponding ansätze 37 and 38 or differentiated ones after the elimination. This can be performed in the function eliminate_lagrangian_multipliers by using the function template replace_all in Table 1.

Last but not least, the template method get_response_functions takes three input parameters as shown in Figure 2. The first two parameters specify which perturbations are to be regarded as respectively extensive and intensive. The last one, min_wfn_exten represents either *k*_*t*_ (for coupled-cluster response theory) or *k*_**D**_ (for AO density-matrix based response theory) that satisfies eq 34 or 36. The minimum order for the elimination of Lagrangian multipliers can be determined as *k*_**λ**_ = max(*k* – *k*_***t***_ + 1, 0) or *k*_**λ**_*a*__ = max(*k* – *k*_**D**_ + 1, 0), which ensures the elimination of differentiated multipliers to the greatest extent.

### 3.2 Task 2: Evaluation of Response Functions

The evaluation of response functions  entails carrying out activities (5) to (9) in Figure 1, where activities (5) and (6) are still performed in a symbolic manner. The former can be straightforwardly carried out by using a function find_all(x, symbol) in Tinned,^23^ which can find the given symbol in differentiated or undifferentiated form in the expression x if they exist there. The latter has been implemented in SymResponse. As shown in Figure 2, the member function get_response_rhs can be used to compute the symbolic expression of the right-hand side of a response equation. Parameters Dw and DP represent respectively **D**_ω_^*b*_1_···*b*_*n*_^ and **D**_P_^*b*_1_···*b*_*n*_^ in eq 19, and rsp_parameter stands for *T̂*_ω_^*b*_1_···*b*_*n*_^ in eq 28 or **λ**_ω_^*b*_1_···*b*_*n*_^ in eq 29 depending on the value of the parameter is_multiplier.

Regarding the AO density-matrix based formulation, we have also developed another member function get_particular_density for computing **K**_ω_^(*n*–1)^ in eq 16, which should be evaluated for the particular solution (14).

In activities (7) to (9), one needs to evaluate different symbolic expressions of—the right-hand side of a response equation, **K**_ω_^(*n*–1)^, and different (un)perturbed electron operators, XC functionals, and nuclear contributions in , etc. Each symbolic expression is represented as a tree-like data structure inside SymEngine,^34^ and can be evaluated by using two template classes in Tinned:^23^FunctionEvaluator and OperatorEvaluator. The former is used to evaluate scalar symbolic expressions such as matrix traces and expectation values, and the latter is for the evaluation of different electron operators. These two template classes provide a way to traverse and evaluate all symbols in the tree-like structure of a symbolic expression, and users need to implement the *helper methods* defined in these two classes.^22^ Each helper method usually evaluates one specific type of symbols, for example, (un)perturbed one- and two-electron operators in the AO density-matrix based formulation, and expectation values of the similarity-transformed Hamiltonian in the coupled-cluster response theory. At the end of the evaluation, the response functions  will be evaluated and returned.

To briefly summarize, the evaluation of response functions—from activity (5) to (9), are actually carried out by users and outside the scope of SymResponse. Users will have full control of the evaluation procedure,^22^ such as evaluating in parallel, and/or caching intermediate results for later use. The evaluation of response functions is not necessarily restricted to be numerical. In the next section, we will show the application of SymResponse for a two-level atom where response functions will be evaluated in a symbolic manner by using the two template classes FunctionEvaluator and OperatorEvaluator.

## 4 Computational Illustration

Thus, far we have presented our unified framework for response theory of two different electronic-structure models. In the current section, we will demonstrate the functionalities of the framework via two elementary examples: the application of response theory for a two-level atom model in which the so-called density operator^24^ is used, and symbolic computations of response functions at the coupled-cluster theory level.

### 4.1 Two-Level Atom

The two-level atom model we choose is adopted from ref (24), and can be described by using the Liouville Equation
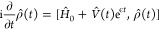
39where the density operator ρ̂(*t*) fulfills both the idempotency constraint ρ̂^2^(*t*) = ρ̂(*t*) and the normalization condition tr[ρ̂(*t*)] = 1. The unperturbed Hamiltonian *Ĥ*_0_ can be written as,^24^
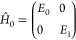
40where *E*_0_ and *E*_1_ are the energy eigenvalues. The external field perturbation *V̂*(*t*) is similar to eq 5,
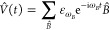
41and the envelope function e^*ϵt*^ has a positive infinitesimal ϵ that ensures a slow switch-on of the field when *t* → – ∞.^24^

Response functions of the two-level atom can also be investigated by using the AO matrix-based response theory.^13^ As shown in Supporting Informationdao_response_orth.pdf, the formulation developed in ref (13) can be directly used by replacing the overlap matrix **S̃** by an identity matrix. In Listing 1, we illustrate how to compute response functions of the two-level atom in a symbolic manner by using SymResponse.^21^
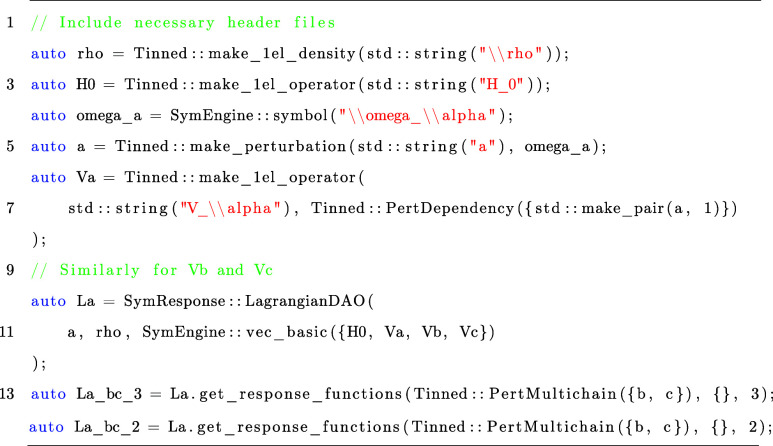


Listing 1: Snippet for computing response functions of the two-level atom by using SymResponse.^21^

The snippet strictly follows activities (1)–(4) in Figure 1 and demonstrates the relative ease with which the SymResponse functionality can be utilized. Lines 2 and 3 create respectively the density operator ρ̂(*t*) and the unperturbed Hamiltonian *Ĥ*_0_ by using functions from Tinned.^23^ In this example, we define three operators *V̂*_α_, *V̂*_β_ and *V̂*_γ_, and each of them depend only on one perturbation differentiating the quasienergy Lagrangian, that is, *a*, *b* and *c*, respectively. Corresponding frequencies are ω_α_, ω_β_ and ω_γ_. Lines 4–9 are used to declare and create these frequencies, perturbations and operators one after another, in which we use the type PertDependency from Tinned to inform the code that each operator can be differentiated only to the first order. After activity (1) performed, lines 10–12 construct *L̃*^*a*^ for activity (2). The last two lines compute response functions  by setting both *b* and *c* as extensive perturbations, but with different elimination rules, i.e., *k*_ρ̂_ [or *k*_**D**_ in eq 36] = 3 and 2, respectively.

One may would like to read results La_bc_3 and La_bc_2 in the form of mathematical expressions. As shown in Table 1, the function latexify from Tinned can convert these results into LATEX that can be compiled to readable expressions. In Supporting Informationtwo_level_atom.pdf, we give the symbolic expressions of La_bc_3 and La_bc_2 by using the function latexify.

Next, we consider the evaluation of the response functions of the two-level atom, in which all operators are represented by corresponding 2 × 2 matrices. For instance, *Ĥ*_0_ is a 2 × 2 diagonal matrix given in eq 40, and the field perturbation *V̂*(*t*) will be provided by users. Recall that the unperturbed system is in the ground state *E*_0_, so the unperturbed or the zeroth-order density operator ρ̂^(0)^ becomes^24^
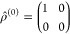
42from which derivatives of the density operator can be evaluated order by order. As shown in Supporting Informationtwo_level_atom.pdf, the *n*-th order derivative of the density operator can be directly obtained from that at the (*n* – 1)-th order, as
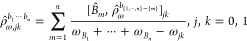
43
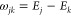
44so that it is not necessary to solve the linear response equation of ρ̂_ω_^*b*_1_···*b*_*n*_^ as that of the AO density matrix-based response theory.^13^

**Figure 3:**
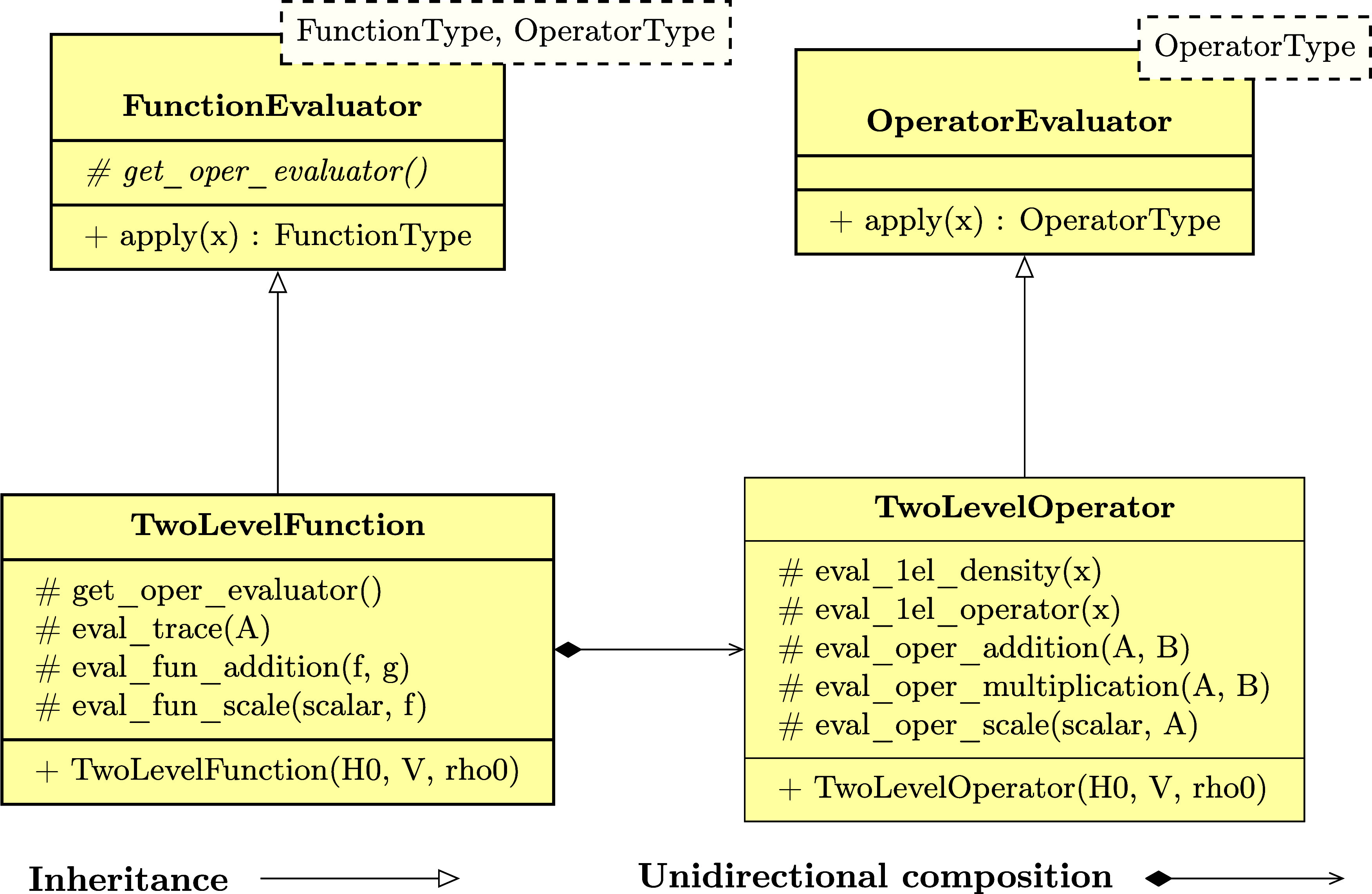
UML class diagram for the evaluation of response functions of the two-level atom.

Our implementation of the evaluation procedure can be summarized in Figure 3. Two subclasses TwoLevelFunction and TwoLevelOperator take care of the evaluation, and inherit respectively from the template classes FunctionEvaluator and OperatorEvaluator. Input parameters of these two subclasses are the same, and contain mapping information between operators and their corresponding 2 × 2 matrices.

Following the subsection **Task 2: evaluation of response functions**, we have overridden a few helper methods (beginning with “eval_” as shown in Figure 3) within these two subclasses that are necessary for the two-level atom’s evaluation. These helper methods can be straightforwardly implemented by using SymEngine,^34^ which provides different symbolic matrix classes and relevant symbolic computations and differentiation. Nevertheless, two helper methods are particularly worth mentioning:

- eval_1el_density(x) evaluates derivatives of the density operator by following eq 43, and for the reason for efficiency, we also cache evaluated derivatives in a C++ map that can be reused in a later stage.
- eval_1el_operator(x) evaluates operators *Ĥ*_0_ and *V̂*, and its algorithm can be described in Listing 2.

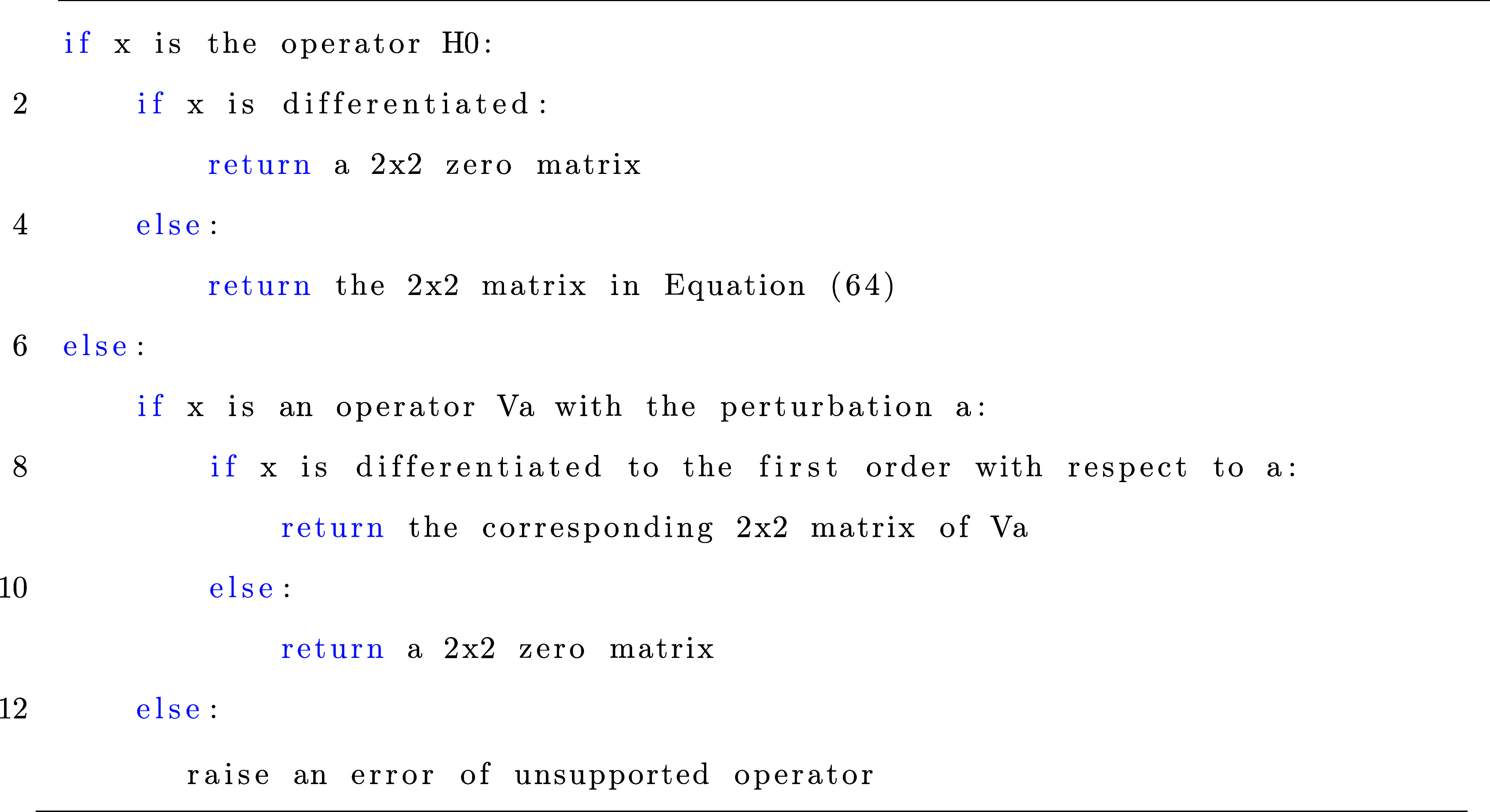


Listing 2: Algorithm for evaluation of *Ĥ*_0_ and *V̂* of the two-level atom.

Last but not least, the template base class FunctionEvaluator also requires an abstract interface get_oper_evaluator to be implemented by subclasses. This interface should return an instance of a subclass of OperatorEvaluator, which will be used for the evaluation of a trace of an operator or a product of several operators. That is, the class FunctionEvaluator will first invoke the instance to evaluate the operator or the product of operators, and then send the evaluated result to the helper method eval_trace(A) for the final evaluation of the trace. Users can certainly use a different strategy for the evaluation of a trace by simply overriding the corresponding function of the class FunctionEvaluator.

The use of the class TwoLevelFunction is rather simple—by calling the function apply with the parameter x being La_bc_3 and La_bc_2 in Listing 1. In Supporting Informationtwo_level_atom.pdf, we give a complete snippet for the evaluation and the final mathematical expressions of these two response functions. It is worth mentioning that, if one categorizes evaluated response functions of the two-level atom according to transition moments in the numerators, there are 12, 54, and 220 unique terms respectively for *L*^*abc*^, the fourth order *L*^*abcd*^ and the fifth order *L*^*abcde*^. It is nevertheless challenging for one to derive their expressions manually.

### 4.2 Symbolic Coupled-Cluster Response Theory Computations

As a final example, we illustrate how one could perform coupled-cluster response theory computations by using SymResponse.^21^ Carrying out this in SymResponse is rather straightforward as shown in Listing 3, where the second- and third-order response functions are computed with three operators Va, Vb and Vc applied.
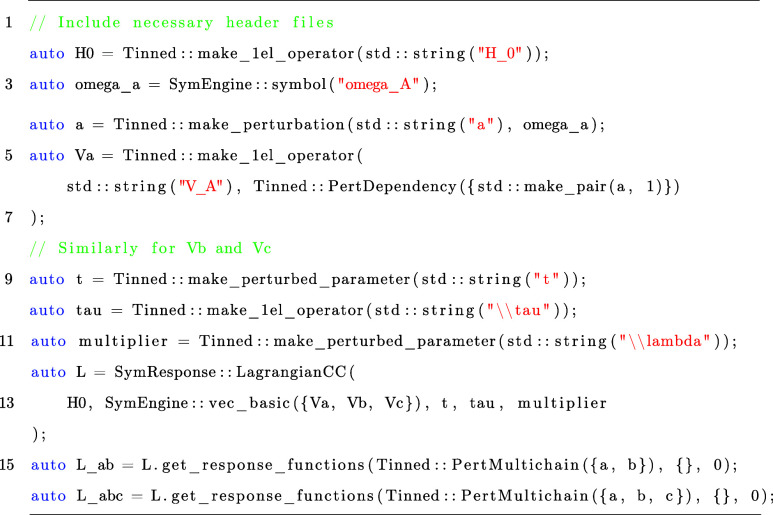


Listing 3: Snippet for computing response functions *L*^*ab*^ and *L*^*abc*^ at the coupled-cluster theory level by using SymResponse.^21^

Similar to the case of the two-level atom, response functions can be straightforwardly obtained by calling the template method get_response_functions after the symbolic representation of the time-averaged quasi-energy Lagrangian *L̃* is created. In the last two lines of Listing 3, we set all perturbations as extensive so that the 2*n* + 1 rule for the coupled-cluster amplitudes ***t*~** and 2*n* + 2 rule for the Lagrangian multipliers **λ̃** are used. The third parameter “0” means that the method get_response_functions will determine the minimum order of differentiated coupled-cluster amplitudes to be eliminated by itself, which is  from eq 34.

In Supporting Informationcc_symbolic_response.pdf, we provide the resulting symbolic expressions of L_ab and L_abc in Listing 3, which we have verified to be equivalent to published results from literature.^11,25,26^

While the actual numerical evaluation of the coupled-cluster response function expressions is outside the scope of the present work, we also demonstrate briefly in this Supporting Information the use of the function get_response_rhs for the Figure 1 activity (6), shown in Listing 4, to identify the constituent terms of the right-hand sides that would need to be evaluated as part of such a calculation. We also remark that the function find_all in Tinned can be used in the Figure 1 activity (5) to search all perturbed amplitudes and multipliers in the computed response functions L_ab and L_abc in Listing 3, which can then be solved one after another with the knowledge of their right-hand sides.
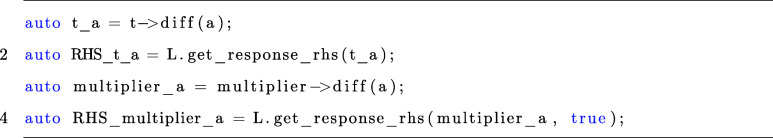


Listing 4: Snippet for computing the right-hand sides of response equations of *t*_ω_^*a*^ and **λ**_ω_^*a*^ by using SymResponse.^21^

In summary, SymResponse provides an opportunity to derive and represent response functions at a symbolic level in a way that we believe can significantly ease the development effort associated with obtaining functionality for response function calculation. The overarching design of SymResponse is general enough to make it readily extensible to other electronic-structure models, such as MCSCF and orbital-relaxed coupled-cluster, at a moderate further investment of SymResponse development effort. Such extensions are considered by us to be relevant topics for future work. Another relevant and relatively straightforward prospective extension of SymResponse is creating routines for determining optimal elimination rules for the calculation of a given property. For instance, one may count the number of perturbed wave function parameters and Lagrangian multipliers to be solved in the symbolic expression of a response function, from which optimal elimination rules may be figured out without the actual (numerical) evaluation.

## 5 Conclusions

We have developed the tool SymResponse for the purpose of facilitating the simulation of molecular response functions of different electronic-structure models. As a demonstration, we have shown the application of SymResponse for the development and computation of AO density matrix-based and coupled-cluster response theories. Extending SymResponse to encompass the response theories associated with further electronic-structure models, such as MCSCF and orbital-relaxed coupled-cluster response theories, is certainly feasible at moderate additional development effort. The underlying design choice in SymResponse that leads to this prospective electronic-structure model versatility can be attributed to the explicit separation between symbolic computations and actual evaluation of response functions, where SymResponse takes care of the symbolic part and the evaluation is left to users. We anticipate that SymResponse will become a useful framework on which to base development of functionality to calculate molecular response properties and intend to apply it ourselves for this purpose in future work.

## Supporting Information Available

cc_response_formulation.pdf: formulation of derivatives of coupled-cluster amplitudes and Lagrangian multipliers by using the introduced notations in the present work (PDF)
dao_response_orth.pdf: density matrix-based quasienergy formulation of the response theory using orthonormal basis sets (PDF)
two_level_atom.pdf: evaluation of response functions of a two-level atom (PDF)
cc_symbolic_response.pdf: symbolic expressions of results from the coupled-cluster response theory computations by using SymResponse^21^ (PDF)
