# Supplementary material for: Unified Framework for Molecular Response Functions of Different Electronic-Structure Models
Source: J Phys Chem A. 2025 Apr 16;129(16):3709–21. doi: 10.1021/acs.jpca.4c07789 (PMC12035861; doi:10.1021/acs.jpca.4c07789)
Supplement: Supplementary file 1 — jp4c07789_si_001.pdf [file jp4c07789_si_001.pdf]

Unified Framework for Molecular Response Functions of Different Electronic-Structure Models

Bin Gao* and Magnus Ringholm

Hylleraas Centre for Quantum Molecular Sciences, Department of Chemistry, UiT The Arctic University of Norway, N–9037 Tromsø, Norway

E-mail: bin.gao@uit.no

In the present Supporting Information, we will recapitulate the formulation of derivatives of the coupled-cluster amplitudes $\boldsymbol{t}_{\omega_{B_1}\cdots\omega_{B_n}}^{b_1\cdots b_n}$ and the Lagrangian multipliers $\boldsymbol{\lambda}_{\omega_{B_1}\cdots\omega_{B_n}}^{b_1\cdots b_n}$ in frequency domain. They can be determined by solving, respectively[1,2]

$$\frac{\partial}{\partial\boldsymbol{\lambda}_{\omega,\mu}^{b_{n+1}\cdots b_{n+m}}}\left(\tilde{L}^{b_1\cdots b_{n+m}}\Big|_{\{\varepsilon\}=0}\right)=0, \tag{S1}$$

and

$$\frac{\partial}{\partial\boldsymbol{t}_{\omega,\nu}^{b_{n+1}\cdots b_{n+m}}}\left(\tilde{L}^{b_1\cdots b_{n+m}}\Big|_{\{\varepsilon\}=0}\right)=0, \tag{S2}$$

where

$$\tilde{L}^{b_1\cdots b_{n+m}}=\frac{\partial^{n+m}\{L(t)\}_T}{\partial\varepsilon_{\omega_{B_1}}\cdots\partial\varepsilon_{\omega_{B_{n+m}}}}, \tag{S3}$$

is the derivative of the time average of the time-dependent coupled-cluster quasi-energy

Lagrangian (20). From Equation (32), we have

$$
\begin{aligned}
\tilde{L}^{b_1\cdots b_{n+m}}\Big|_{\{\varepsilon\}=0} &= \langle \Big[\mathrm{e}^{\mathrm{ad}_{-\hat{T}(t)}}(\hat{H}(t))\Big]_{\omega}^{b_1\cdots b_{n+m}} \rangle \\
&+ \sum_{\substack{P\subseteq\{1,\cdots,n+m\}\\ Q=\{1,\cdots,n+m\}-P}} \boldsymbol{\lambda}_{\omega,\mu}^{b_P} \langle \Big[\mathrm{e}^{\mathrm{ad}_{-\hat{T}(t)}}(\hat{H}(t))\Big]_{\omega}^{b_Q} \rangle_{\mu} \\
&- \sum_{\substack{P\subseteq\{1,\cdots,n+m\}\\ Q=\{1,\cdots,n+m\}-P}} \boldsymbol{\lambda}_{\omega,\mu}^{b_P} \left(\sum_{q\in Q}\omega_{B_q}\right) \boldsymbol{t}_{\omega,\mu}^{b_Q},
\end{aligned} \tag{S4}
$$

where the perturbation-strength derivatives of $\mathrm{e}^{\mathrm{ad}_{-\hat{T}(t)}}(\hat{H}(t))$ can be expressed as[3]

$$
\begin{aligned}
\Big[\mathrm{e}^{\mathrm{ad}_{-\hat{T}(t)}}(\hat{H}(t))\Big]_{\omega}^{b_1\cdots b_n} &= \mathrm{e}^{\mathrm{ad}_{-\hat{T}(0)}}(\hat{H}_{\omega}^{b_1\cdots b_n}) \\
&+ \sum_{\substack{P\subsetneq\{1,\cdots,n\}\\ Q=\{1,\cdots,n\}-P\\ |Q|\geq 4}} \sum_{\pi\in\mathcal{P}_{Q,4}} \prod_{q=1}^{4} (\mathrm{ad}_{\hat{T}_{\omega}^{b_{\pi_q}}})(\hat{H}_{\omega}^{b_P}) \\
&+ \sum_{k=1}^{3} \sum_{\substack{P\subsetneq\{1,\cdots,n\}\\ Q=\{1,\cdots,n\}-P\\ |Q|\geq k}} \sum_{\pi\in\mathcal{P}_{Q,k}} (-1)^k \mathrm{e}^{\mathrm{ad}_{-\hat{T}(0)}}(\prod_{q=1}^{k}(\mathrm{ad}_{\hat{T}_{\omega}^{b_{\pi_q}}})(\hat{H}_{\omega}^{b_P})),
\end{aligned} \tag{S5}
$$

with

$$
\hat{H}_{\omega}^{b_P} = \hat{H}(t)^{b_P}\Big|_{\{\varepsilon\}=0}, \tag{S6}
$$

$$
\hat{T}_{\omega}^{b_{\pi_q}} = \sum_{\nu} \boldsymbol{t}_{\omega,\nu}^{b_{\pi_q}} \hat{\boldsymbol{\tau}}_{\nu}. \tag{S7}
$$

The response equation of the nth-order derivative of the coupled-cluster amplitudes $\boldsymbol{t}_{\omega_{B_1}\cdots\omega_{B_n}}^{b_1\cdots b_n}$ can thus be obtained from Equation (S1) and only the last two terms in Equation (S4) with the sets of perturbation indices $P = \{n+1,\cdots,n+m\}$ and $Q = \{1,\cdots,n\}$ can survive. By collecting all lower-order derivatives of the coupled-cluster amplitudes on

the right-hand side, the response equation reads

$$(\mathbf{A}-\omega_{B_N}\mathbf{I})\,\boldsymbol{t}^{b_1\cdots b_n,\mathrm{T}}_{\omega_{B_1}\cdots\omega_{B_n}}=-\boldsymbol{\xi}^{b_1\cdots b_n,\mathrm{T}}_{\omega},\tag{S8}$$

where the superscript "T" denotes the transposition of row vectors, the sum of frequencies ω_{B_N} is defined in Equation (18), $\mathbf{I}$ is an $n\times n$ identity matrix and $\mathbf{A}$ is the nonsymmetric coupled-cluster Jacobian[1,4]

$$\mathbf{A}_{\mu\nu}=\langle \mathrm{e}^{-\hat{T}^{(0)}}[\hat{H}_0,\hat{\boldsymbol{\tau}}_\nu]\mathrm{e}^{\hat{T}^{(0)}}\rangle_\mu=\langle \mathrm{e}^{\mathrm{ad}_{-\hat{T}^{(0)}}}([\hat{H}_0,\hat{\boldsymbol{\tau}}_\nu])\rangle_\mu,\tag{S9}$$

where

$$\hat{T}^{(0)}=\sum_\nu \boldsymbol{t}^{(0)}_\nu\hat{\boldsymbol{\tau}}_\nu,\tag{S10}$$

with $\boldsymbol{t}^{(0)}$ the zeroth-order coupled-cluster amplitudes.

As aforementioned, the right-hand side vector $\boldsymbol{\xi}^{b_1\cdots b_n}_{\omega}$ contains all lower-order derivatives of the coupled-cluster amplitudes of Equation (S5), and can be written compactly by using the notations and definitions of the present work as

$$\begin{aligned}\boldsymbol{\xi}^{b_1\cdots b_n}_{\omega,\mu}&=\langle\left[\mathrm{e}^{\mathrm{ad}_{-\hat{T}(t)}}(\hat{H}(t))\right]^{b_1\cdots b_n}_{\omega}\Bigg|_{\substack{\text{Remove terms}\\ \text{involving }\hat{T}^{b_1\cdots b_n}_{\omega}}}\rangle_\mu=\langle \mathrm{e}^{\mathrm{ad}_{-\hat{T}^{(0)}}}(\hat{H}^{b_1\cdots b_n}_{\omega})\rangle_\mu\\ &+\sum_{\substack{P\subsetneq\{1,\cdots,n\}\\ Q=\{1,\cdots,n\}-P\\ |Q|\geq 4}}\sum_{\pi\in\mathcal{P}_{Q,4}}\langle\prod_{q=1}^{4}(\mathrm{ad}_{\hat{\boldsymbol{\tau}}_{\nu_q}})(\hat{H}^{b_P}_{\omega})\rangle_\mu\bigotimes_{q=1}^{4}\boldsymbol{t}^{b_{\pi_q}}_{\omega,\nu_q}\\ &+\sum_{k=2}^{3}\sum_{\substack{P\subsetneq\{1,\cdots,n\}\\ Q=\{1,\cdots,n\}-P\\ |Q|\geq k}}\sum_{\pi\in\mathcal{P}_{Q,k}}(-1)^k\langle \mathrm{e}^{\mathrm{ad}_{-\hat{T}^{(0)}}}(\prod_{q=1}^{k}(\mathrm{ad}_{\hat{\boldsymbol{\tau}}_{\nu_q}})(\hat{H}^{b_P}_{\omega}))\rangle_\mu\bigotimes_{q=1}^{k}\boldsymbol{t}^{b_{\pi_q}}_{\omega,\nu_q}\\ &-\sum_{\substack{P\subsetneq\{1,\cdots,n\}\\ Q=\{1,\cdots,n\}-P\\ |P|\geq 1,\,|Q|\geq 1}}\langle \mathrm{e}^{\mathrm{ad}_{-\hat{T}^{(0)}}}(\mathrm{ad}_{\hat{\boldsymbol{\tau}}_{\nu_1}}(\hat{H}^{b_P}_{\omega}))\rangle_\mu\boldsymbol{t}^{b_Q}_{\omega,\nu_1},\end{aligned}\tag{S11}$$

where $\otimes$ stands for tensor products and the Einstein summation convention has been used

for $\nu_1, \cdots, \nu_4$.

The response equation for the Lagrangian multipliers can be obtained in a similar manner. We first need to find which terms containing $\boldsymbol{t}_{\omega,\nu}^{b_{n+1}\cdots b_{n+m}}$ (or $\hat{T}_{\omega}^{b_{n+1}\cdots b_{n+m}}$) in $\tilde{L}^{b_1\cdots b_{n+m}}\Big|_{\{\varepsilon\}=0}$ that will survive after the differentiation in Equation (S2). For any $Q \subseteq \{1, \cdots, n\}$, terms involving $\hat{T}_{\omega}^{b_{n+1}\cdots b_{n+m}}$ of $\left[\mathrm{e}^{\mathrm{ad}_{-\hat{T}(t)}}(\hat{H}(t))\right]_{\omega}^{b_{Q\cup\{n+1,\cdots,n+m\}}}$ can be obtained by using Equation (S5), as

$$\left[\mathrm{e}^{\mathrm{ad}_{-\hat{T}(t)}}(\hat{H}(t))\right]_{\omega}^{b_{Q\cup\{n+1,\cdots,n+m\}}}\Bigg|_{\substack{\text{Remove terms}\\ \text{not involving } \hat{T}_{\omega}^{b_{n+1}\cdots b_{n+m}}}} = \mathrm{e}^{\mathrm{ad}_{-\hat{T}^{(0)}}}([\hat{H}_{\omega}^{b_Q}, \hat{T}_{\omega}^{b_{n+1}\cdots b_{n+m}}])$$
$$- \sum_{\substack{R\subsetneq Q\\ S=Q-R\\ |S|\geq 3}} \sum_{\pi\in\mathcal{P}_{S,3}} \prod_{s=1}^{3}(\mathrm{ad}_{\hat{T}_{\omega}^{b_{\pi_s}}})([\hat{H}_{\omega}^{b_R}, \hat{T}_{\omega}^{b_{n+1}\cdots b_{n+m}}])$$
$$+ \sum_{k=1}^{2} \sum_{\substack{R\subsetneq Q\\ S=Q-R\\ |S|\geq k}} \sum_{\pi\in\mathcal{P}_{S,k}} (-1)^k \mathrm{e}^{\mathrm{ad}_{-\hat{T}^{(0)}}}(\prod_{s=1}^{k}(\mathrm{ad}_{\hat{T}_{\omega}^{b_{\pi_s}}})([\hat{H}_{\omega}^{b_R}, \hat{T}_{\omega}^{b_{n+1}\cdots b_{n+m}}])). \quad \text{(S12)}$$

From the above equation and noting that the last term of Equation (S4) has only the following non-zero contribution,

$$-\boldsymbol{\lambda}_{\omega,\nu}^{b_1\cdots b_n}\left(\sum_{j=n+1}^{n+m}\omega_{B_j}\right) = \boldsymbol{\lambda}_{\omega,\nu}^{b_1\cdots b_n}\left(\sum_{j=1}^{n}\omega_{B_j}\right), \quad \text{(S13)}$$

we get the following response equation for the Lagrangian multipliers after taking the derivatives with respect to $\boldsymbol{t}_{\omega,\nu}^{b_{n+1}\cdots b_{n+m}}$ in Equation (S2),

$$\boldsymbol{\lambda}_{\omega_{B_1}\cdots\omega_{B_n}}^{b_1\cdots b_n}(\mathbf{A} + \omega_{B_N}\mathbf{I}) = -\boldsymbol{\zeta}_{\omega}^{b_1\cdots b_n}, \quad \text{(S14)}$$

where the right-hand side $\boldsymbol{\zeta}_{\omega}^{b_1\cdots b_n}$ is obtained by collecting lower-order derivatives of the

Lagrangian multipliers after the differentiation of Equation (S2),

$$\begin{aligned}
\boldsymbol{\zeta}_{\omega,\nu}^{b_1\cdots b_n} &= \langle \left[\mathrm{e}^{\mathrm{ad}_{-\hat{T}(t)}}([\hat{H}(t), \hat{\boldsymbol{\tau}}_\nu]) \right]_{\omega}^{b_1\cdots b_n} \rangle + \left[\tilde{\boldsymbol{\lambda}}_\mu \langle \mathrm{e}^{\mathrm{ad}_{-\hat{T}(t)}}([\hat{H}(t), \hat{\boldsymbol{\tau}}_\nu]) \rangle_\mu \right]_{\omega}^{b_1\cdots b_n} \Bigg|_{\substack{\text{Remove terms} \\ \text{involving } \boldsymbol{\lambda}_{\omega,\mu}^{b_1\cdots b_n}}} \\
&= \langle \mathrm{e}^{\mathrm{ad}_{-\hat{T}^{(0)}}}([\hat{H}_\omega^{b_1\cdots b_n}, \hat{\boldsymbol{\tau}}_\nu]) \rangle \\
&\quad - \sum_{\substack{P \subsetneq \{1,\cdots,n\} \\ Q=\{1,\cdots,n\}-P \\ |Q|\geq 3}} \sum_{\pi \in \mathcal{P}_{Q,3}} \langle \prod_{q=1}^{3} (\mathrm{ad}_{\hat{\boldsymbol{\tau}}_{\nu_q}})([\hat{H}_\omega^{b_P}, \hat{\boldsymbol{\tau}}_\nu]) \rangle \bigotimes_{q=1}^{3} \boldsymbol{t}_{\omega,\nu_q}^{b_{\pi_q}} \\
&\quad + \sum_{k=1}^{2} \sum_{\substack{P \subsetneq \{1,\cdots,n\} \\ Q=\{1,\cdots,n\}-P \\ |Q|\geq k}} \sum_{\pi \in \mathcal{P}_{Q,k}} (-1)^k \langle \mathrm{e}^{\mathrm{ad}_{-\hat{T}^{(0)}}} (\prod_{q=1}^{k} (\mathrm{ad}_{\hat{\boldsymbol{\tau}}_{\nu_q}})([\hat{H}_\omega^{b_P}, \hat{\boldsymbol{\tau}}_\nu])) \rangle \bigotimes_{q=1}^{k} \boldsymbol{t}_{\omega,\nu_q}^{b_{\pi_q}} \\
&\quad + \sum_{\substack{P \subsetneq \{1,\cdots,n\} \\ Q=\{1,\cdots,n\}-P}} \boldsymbol{\lambda}_{\omega,\mu}^{b_P} \langle \mathrm{e}^{\mathrm{ad}_{-\hat{T}^{(0)}}}([\hat{H}_\omega^{b_Q}, \hat{\boldsymbol{\tau}}_\nu]) \rangle_\mu \\
&\quad - \sum_{\substack{P \subsetneq \{1,\cdots,n\} \\ Q=\{1,\cdots,n\}-P}} \sum_{\substack{R \subsetneq Q \\ S=Q-R \\ |S|\geq 3}} \sum_{\pi \in \mathcal{P}_{S,3}} \boldsymbol{\lambda}_{\omega,\mu}^{b_P} \langle \prod_{s=1}^{3} (\mathrm{ad}_{\hat{\boldsymbol{\tau}}_{\nu_s}})([\hat{H}_\omega^{b_R}, \hat{\boldsymbol{\tau}}_\nu]) \rangle_\mu \bigotimes_{s=1}^{3} \boldsymbol{t}_{\omega,\nu_s}^{b_{\pi_s}} \\
&\quad + \sum_{\substack{P \subsetneq \{1,\cdots,n\} \\ Q=\{1,\cdots,n\}-P}} \sum_{k=1}^{2} \sum_{\substack{R \subsetneq Q \\ S=Q-R \\ |S|\geq k}} \sum_{\pi \in \mathcal{P}_{S,k}} (-1)^k \boldsymbol{\lambda}_{\omega,\mu}^{b_P} \langle \mathrm{e}^{\mathrm{ad}_{-\hat{T}^{(0)}}} (\prod_{s=1}^{k} (\mathrm{ad}_{\hat{\boldsymbol{\tau}}_{\nu_s}})([\hat{H}_\omega^{b_R}, \hat{\boldsymbol{\tau}}_\nu])) \rangle_\mu \bigotimes_{s=1}^{k} \boldsymbol{t}_{\omega,\nu_s}^{b_{\pi_s}},
\end{aligned} \tag{S15}$$

with the Einstein summation convention for $\nu_1, \cdots, \nu_4$.

References

(1) Hättig, C.; Christiansen, O.; Jørgensen, P. Multiphoton transition moments and absorption cross sections in coupled cluster response theory employing variational transition moment functionals. *J. Chem. Phys.* **1998**, *108*, 8331–8354.

(2) Pedersen, T. B.; Koch, H. Coupled cluster response functions revisited. *J. Chem. Phys.* **1997**, *106*, 8059–8072.

(3) Gao, B. Tinned: A symbolic library for response theory and high-order derivatives. *J. Comput. Chem.* **2024**, *45*, 2136–2152.

(4) Helgaker, T.; Coriani, S.; Jørgensen, P.; Kristensen, K.; Olsen, J.; Ruud, K. Recent Advances in Wave Function-Based Methods of Molecular-Property Calculations. *Chem. Rev.* **2012**, *112*, 543–631.
