# Supplementary material for: Unified Framework for Molecular Response Functions of Different Electronic-Structure Models
Source: J Phys Chem A. 2025 Apr 16;129(16):3709–21. doi: 10.1021/acs.jpca.4c07789 (PMC12035861; doi:10.1021/acs.jpca.4c07789)
Supplement: Supplementary file 2 — jp4c07789_si_002.pdf [file jp4c07789_si_002.pdf]

Unified Framework for Molecular Response Functions of Different Electronic-Structure Models

Bin Gao* and Magnus Ringholm

Hylleraas Centre for Quantum Molecular Sciences, Department of Chemistry, UiT The Arctic University of Norway, N–9037 Tromsø, Norway

E-mail: bin.gao@uit.no

The objective of the current supporting information is to derive the variational time-averaged quasienergy derivative Lagrangian $\tilde{L}^a$ when orthonormal basis sets are used. Our starting point is Equation (54) of Reference 1, i.e., the quasienergy Lagrangian in the molecular orbital (MO) coefficient matrix $\tilde{\mathbf{C}}$ representation,

$$\tilde{L}(\tilde{\mathbf{C}}, \tilde{\boldsymbol{\lambda}}, t) = \tilde{E}(\tilde{\mathbf{C}}, t) - \mathrm{i}\sum_{I}\langle\tilde{\phi}_I|\dot{\tilde{\phi}}_I\rangle - \sum_{IJ}\tilde{\boldsymbol{\lambda}}_{JI}(\langle\tilde{\phi}_I|\tilde{\phi}_j\rangle - \delta_{IJ}). \tag{S1}$$

For orthonormal basis sets, the overlap matrix $\tilde{\mathbf{S}}$ becomes an identity matrix $\mathbf{I}$, and the orthonormality condition for the occupied MOs is then,

$$\boldsymbol{\rho}\tilde{\mathbf{C}}^{\dagger}\tilde{\mathbf{C}}\boldsymbol{\rho} = \boldsymbol{\rho}, \tag{S2}$$

with $\boldsymbol{\rho}$ the MO density matrix defined in Equation (40) of Reference 1. So the second and

third terms of $\tilde{L}(\tilde{\mathbf{C}}, \tilde{\boldsymbol{\lambda}}, t)$ can be written as

$$-\mathrm{i}\sum_I \langle \tilde{\phi}_I | \dot{\tilde{\phi}}_I \rangle \overset{\mathrm{tr}}{=} -\mathrm{i}(\boldsymbol{\rho}\tilde{\mathbf{C}}^\dagger \tilde{\mathbf{R}} \tilde{\mathbf{C}} \boldsymbol{\rho} + \boldsymbol{\rho}\tilde{\mathbf{C}}^\dagger \dot{\tilde{\mathbf{C}}} \boldsymbol{\rho}), \tag{S3}$$

$$-\sum_{IJ} \tilde{\boldsymbol{\lambda}}_{JI}(\langle \tilde{\phi}_I | \tilde{\phi}_j \rangle - \delta_{IJ}) \overset{\mathrm{tr}}{=} -(\tilde{\mathbf{C}}^\dagger \tilde{\mathbf{C}} \boldsymbol{\rho} \tilde{\boldsymbol{\lambda}} \boldsymbol{\rho} - \boldsymbol{\rho} \tilde{\boldsymbol{\lambda}} \boldsymbol{\rho}), \tag{S4}$$

where the notation $\overset{\mathrm{tr}}{=}$ denotes a trace of matrix products on the right-hand side, and $\tilde{\mathbf{R}}_{\mu\nu} = \langle \tilde{\chi}_\mu | \dot{\tilde{\chi}}_\nu \rangle$.

Notice that differentiated $\tilde{\mathbf{C}}^a$ and $\tilde{\boldsymbol{\lambda}}^a$ do not contribute to the perturbation-strength derivative of the time-averaged quasienergy Lagrangian,[1] and

$$\tilde{\mathbf{D}} = \tilde{\mathbf{C}} \boldsymbol{\rho} \tilde{\mathbf{C}}^\dagger, \tag{S5}$$

we have

$$\frac{\partial}{\partial \varepsilon_a}\left(-\mathrm{i}\sum_I \langle \tilde{\phi}_I | \dot{\tilde{\phi}}_I \rangle - \sum_{IJ} \tilde{\boldsymbol{\lambda}}_{JI}(\langle \tilde{\phi}_I | \tilde{\phi}_j \rangle - \delta_{IJ})\right) \overset{\mathrm{tr}}{=} -\mathrm{i}\tilde{\mathbf{R}}^a \tilde{\mathbf{D}} \overset{\mathrm{tr}}{=} -\frac{\mathrm{i}}{2}\tilde{\mathbf{T}}^a \tilde{\mathbf{D}}, \tag{S6}$$

where, by noticing $\dot{\tilde{\mathbf{S}}} = \tilde{\mathbf{R}} + \tilde{\mathbf{R}}^\dagger = \mathbf{0}$,

$$\tilde{\mathbf{T}} = \tilde{\mathbf{R}} - \tilde{\mathbf{R}}^\dagger = -2\tilde{\mathbf{R}} \overset{\mathrm{or}}{=} 2\tilde{\mathbf{R}}^\dagger. \tag{S7}$$

Therefore, the perturbation-strength derivative of the time-averaged Equation (S1) can be written in the density matrix $\tilde{\mathbf{D}}$ representation as,

$$\tilde{L}^a(\tilde{\mathbf{C}}, \tilde{\boldsymbol{\lambda}}) \equiv \tilde{E}^{0,a}, \tag{S8}$$

with the generalized energy $\tilde{E}$ being

$$\tilde{E} \overset{\{\mathrm{tr}\}_T}{=} \left[\tilde{\mathbf{h}} + \tilde{\mathbf{V}} + \frac{1}{2}\tilde{\mathbf{G}}^\gamma(\tilde{\mathbf{D}}) - \frac{\mathrm{i}}{2}\tilde{\mathbf{T}}\right]\tilde{\mathbf{D}} + \tilde{E}_{\mathrm{xc}}[\tilde{\rho}(\tilde{\mathbf{D}})] + \tilde{h}_{\mathrm{nuc}}. \tag{S9}$$

The difference between Equation (S8) and Equation (98) of Reference 1 is the absence of the Pulay term due to the use of orthonormal basis sets.

Now we can write the time-averaged quasienergy derivative Lagrangian in terms of the density matrix $\tilde{\mathbf{D}}$,

$$\tilde{L}^a(\tilde{\mathbf{D}}, \tilde{\boldsymbol{\lambda}}_a, \tilde{\boldsymbol{\zeta}}_a) \overset{\{\mathrm{tr}\}_T}{=} \tilde{E}^{0,a} - \tilde{\boldsymbol{\lambda}}_a \tilde{\mathbf{Y}} - \tilde{\boldsymbol{\zeta}}_a \tilde{\mathbf{Z}}, \tag{S10}$$

where the time-dependent self-consistent-field (TDSCF) equation $\tilde{\mathbf{Y}}$ can be obtained by following the same procedure as Equation (106) of Reference 1,

$$\tilde{\mathbf{Y}} = \tilde{\mathbf{F}}\tilde{\mathbf{D}} - \tilde{\mathbf{D}}\tilde{\mathbf{F}} - \mathrm{i}\frac{\partial \tilde{\mathbf{D}}}{\partial t}, \tag{S11}$$

with the generalized Fock matrix as

$$\tilde{\mathbf{F}} = \tilde{\mathbf{h}} + \tilde{\mathbf{V}} + \tilde{\mathbf{G}}^{\gamma}(\tilde{\mathbf{D}}) + \tilde{\mathbf{F}}_{\mathrm{xc}}(\tilde{\rho}) - \frac{\mathrm{i}}{2}\tilde{\mathbf{T}}. \tag{S12}$$

The idempotency constraint $\tilde{\mathbf{Z}}$ can be obtained by multiplying Equation (S2) by $\tilde{\mathbf{C}}$ from the left and $\tilde{\mathbf{C}}^{\dagger}$ from the right, and using Equation (S5),

$$\tilde{\mathbf{Z}} = \tilde{\mathbf{D}}\tilde{\mathbf{D}} - \tilde{\mathbf{D}}. \tag{S13}$$

Finally, we will show that $\tilde{L}^a(\tilde{\mathbf{D}}, \tilde{\boldsymbol{\lambda}}_a, \tilde{\boldsymbol{\zeta}}_a)$ is variational with respect to $\tilde{\mathbf{D}}$,

$$\frac{\partial}{\partial \tilde{\mathbf{D}}^{\mathrm{T}}} \tilde{L}^a(\tilde{\mathbf{D}}, \tilde{\boldsymbol{\lambda}}_a, \tilde{\boldsymbol{\zeta}}_a) = \mathbf{0}, \tag{S14}$$

by choosing the following ansatzes for Lagrangian multipliers $\tilde{\boldsymbol{\lambda}}_a$ and $\tilde{\boldsymbol{\zeta}}_a$

$$\tilde{\boldsymbol{\lambda}}_a = \tilde{\mathbf{D}}^a\tilde{\mathbf{D}} - \tilde{\mathbf{D}}\tilde{\mathbf{D}}^a, \tag{S15}$$

$$\tilde{\boldsymbol{\zeta}}_a = \tilde{\mathbf{F}}^a\tilde{\mathbf{D}} + \tilde{\mathbf{D}}\tilde{\mathbf{F}}^a - \tilde{\mathbf{F}}^a. \tag{S16}$$

These ansatzes are respectively from Equations (220) and (224) of Reference 1 by simply making $\tilde{\mathbf{S}} = \mathbf{I}$, $\frac{\partial}{\partial t}\tilde{\mathbf{S}} = \mathbf{0}$ and $\tilde{\mathbf{S}}^a = \mathbf{0}$.

To prove Equation (S14), we first have

$$\frac{\partial}{\partial \tilde{\mathbf{D}}^{\mathrm{T}}} \tilde{L}^a(\tilde{\mathbf{D}}, \tilde{\boldsymbol{\lambda}}_a, \tilde{\boldsymbol{\zeta}}_a) = \tilde{E}^{1,a} + \tilde{E}^2(\tilde{\boldsymbol{\lambda}}_a\tilde{\mathbf{D}} - \tilde{\mathbf{D}}\tilde{\boldsymbol{\lambda}}_a) + \tilde{\mathbf{F}}\tilde{\boldsymbol{\lambda}}_a - \tilde{\boldsymbol{\lambda}}_a\tilde{\mathbf{F}} - \mathrm{i}\frac{\partial \tilde{\boldsymbol{\lambda}}_a}{\partial t} - \tilde{\boldsymbol{\zeta}}_a\tilde{\mathbf{D}} - \tilde{\mathbf{D}}\tilde{\boldsymbol{\zeta}}_a + \tilde{\boldsymbol{\zeta}}_a, \quad \text{(S17)}$$

by using[1]

$$\frac{\partial}{\partial \tilde{\mathbf{D}}^{\mathrm{T}}} \mathrm{tr}(\tilde{\mathbf{D}}\tilde{\mathbf{A}}) = \tilde{\mathbf{A}}, \quad \text{(S18)}$$

$$\frac{\partial}{\partial \tilde{\mathbf{D}}^{\mathrm{T}}} \mathrm{tr}(\tilde{\mathbf{F}}\tilde{\mathbf{M}}) = \tilde{E}^2(\tilde{\mathbf{M}}), \quad \text{(S19)}$$

$$\left\{ \mathrm{tr}\left[\frac{\partial}{\partial t}(\tilde{\mathbf{D}}\tilde{\boldsymbol{\lambda}}_a) \right] \right\}_T = 0. \quad \text{(S20)}$$

The sum of the first two terms of Equation (S17) is

$$\tilde{E}^{1,a} + \tilde{E}^2(\tilde{\boldsymbol{\lambda}}_a\tilde{\mathbf{D}} - \tilde{\mathbf{D}}\tilde{\boldsymbol{\lambda}}_a) = \tilde{E}^{1,a} + \tilde{E}^2(\tilde{\mathbf{D}}^a) = \tilde{\mathbf{F}}^a, \quad \text{(S21)}$$

by noticing

$$\begin{aligned} \tilde{\boldsymbol{\lambda}}_a\tilde{\mathbf{D}} - \tilde{\mathbf{D}}\tilde{\boldsymbol{\lambda}}_a &= (\tilde{\mathbf{D}}^a\tilde{\mathbf{D}} - \tilde{\mathbf{D}}\tilde{\mathbf{D}}^a)\tilde{\mathbf{D}} - \tilde{\mathbf{D}}(\tilde{\mathbf{D}}^a\tilde{\mathbf{D}} - \tilde{\mathbf{D}}\tilde{\mathbf{D}}^a) \\ &= (2\tilde{\mathbf{D}}^a\tilde{\mathbf{D}} - \tilde{\mathbf{D}}^a)\tilde{\mathbf{D}} - \tilde{\mathbf{D}}(\tilde{\mathbf{D}}^a - 2\tilde{\mathbf{D}}\tilde{\mathbf{D}}^a) \\ &= 2\tilde{\mathbf{D}}^a\tilde{\mathbf{D}} - \tilde{\mathbf{D}}^a\tilde{\mathbf{D}} - \tilde{\mathbf{D}}\tilde{\mathbf{D}}^a + 2\tilde{\mathbf{D}}\tilde{\mathbf{D}}^a = \tilde{\mathbf{D}}^a, \end{aligned} \quad \text{(S22)}$$

where $\tilde{\mathbf{Z}} = \tilde{\mathbf{D}}\tilde{\mathbf{D}} - \tilde{\mathbf{D}} = \mathbf{0}$ and $\tilde{\mathbf{Z}}^a = \tilde{\mathbf{D}}^a\tilde{\mathbf{D}} + \tilde{\mathbf{D}}\tilde{\mathbf{D}}^a - \tilde{\mathbf{D}}^a = \mathbf{0}$ have been used.

Next, we consider the fifth term of Equation (S17), which becomes

$$
\begin{aligned}
-\mathrm{i}\frac{\partial\tilde{\boldsymbol{\lambda}}_a}{\partial t} &= -\mathrm{i}\frac{\partial}{\partial t}(\tilde{\mathbf{D}}^a\tilde{\mathbf{D}}-\tilde{\mathbf{D}}\tilde{\mathbf{D}}^a) = -\mathrm{i}\frac{\partial\tilde{\mathbf{D}}^a}{\partial t}\tilde{\mathbf{D}}-\mathrm{i}\tilde{\mathbf{D}}^a\frac{\partial\tilde{\mathbf{D}}}{\partial t}+\mathrm{i}\frac{\partial\tilde{\mathbf{D}}}{\partial t}\tilde{\mathbf{D}}^a+\mathrm{i}\tilde{\mathbf{D}}\frac{\partial\tilde{\mathbf{D}}^a}{\partial t} \\
&= -(\tilde{\mathbf{F}}^a\tilde{\mathbf{D}}+\tilde{\mathbf{F}}\tilde{\mathbf{D}}^a-\tilde{\mathbf{D}}^a\tilde{\mathbf{F}}-\tilde{\mathbf{D}}\tilde{\mathbf{F}}^a)\tilde{\mathbf{D}}-\tilde{\mathbf{D}}^a(\tilde{\mathbf{F}}\tilde{\mathbf{D}}-\tilde{\mathbf{D}}\tilde{\mathbf{F}}) \\
&+(\tilde{\mathbf{F}}\tilde{\mathbf{D}}-\tilde{\mathbf{D}}\tilde{\mathbf{F}})\tilde{\mathbf{D}}^a+\tilde{\mathbf{D}}(\tilde{\mathbf{F}}^a\tilde{\mathbf{D}}+\tilde{\mathbf{F}}\tilde{\mathbf{D}}^a-\tilde{\mathbf{D}}^a\tilde{\mathbf{F}}-\tilde{\mathbf{D}}\tilde{\mathbf{F}}^a) \\
&= -\tilde{\mathbf{F}}^a\tilde{\mathbf{D}}-\tilde{\mathbf{D}}\tilde{\mathbf{F}}^a-\tilde{\mathbf{F}}(\tilde{\mathbf{D}}^a\tilde{\mathbf{D}}-\tilde{\mathbf{D}}\tilde{\mathbf{D}}^a)+(\tilde{\mathbf{D}}^a\tilde{\mathbf{D}}-\tilde{\mathbf{D}}\tilde{\mathbf{D}}^a)\tilde{\mathbf{F}}+2\tilde{\mathbf{D}}\tilde{\mathbf{F}}^a\tilde{\mathbf{D}} \\
&= -\tilde{\mathbf{F}}^a-\tilde{\boldsymbol{\zeta}}_a-\tilde{\mathbf{F}}\tilde{\boldsymbol{\lambda}}_a+\tilde{\boldsymbol{\lambda}}_a\tilde{\mathbf{F}}+2\tilde{\mathbf{D}}\tilde{\mathbf{F}}^a\tilde{\mathbf{D}}, \qquad \text{(S23)}
\end{aligned}
$$

by using Equations (S15), (S16), $\tilde{\mathbf{Z}}=\mathbf{0}$, $\tilde{\mathbf{Y}}=\mathbf{0}$, and

$$
\tilde{\mathbf{Y}}^a = \tilde{\mathbf{F}}^a\tilde{\mathbf{D}}+\tilde{\mathbf{F}}\tilde{\mathbf{D}}^a-\tilde{\mathbf{D}}^a\tilde{\mathbf{F}}-\tilde{\mathbf{D}}\tilde{\mathbf{F}}^a-\mathrm{i}\frac{\partial\tilde{\mathbf{D}}^a}{\partial t}=\mathbf{0}. \qquad \text{(S24)}
$$

Last, by substituting Equations (S15), (S16), (S21) and (S23) into (S17), we get

$$
\begin{aligned}
\frac{\partial}{\partial\tilde{\mathbf{D}}^{\mathrm{T}}}\tilde{L}^a(\tilde{\mathbf{D}},\tilde{\boldsymbol{\lambda}}_a,\tilde{\boldsymbol{\zeta}}_a) &= \tilde{\mathbf{F}}^a+\tilde{\mathbf{F}}\tilde{\boldsymbol{\lambda}}_a-\tilde{\boldsymbol{\lambda}}_a\tilde{\mathbf{F}}-\tilde{\mathbf{F}}^a-\tilde{\boldsymbol{\zeta}}_a-\tilde{\mathbf{F}}\tilde{\boldsymbol{\lambda}}_a+\tilde{\boldsymbol{\lambda}}_a\tilde{\mathbf{F}}+2\tilde{\mathbf{D}}\tilde{\mathbf{F}}^a\tilde{\mathbf{D}} \\
&-(\tilde{\mathbf{F}}^a\tilde{\mathbf{D}}+\tilde{\mathbf{D}}\tilde{\mathbf{F}}^a-\tilde{\mathbf{F}}^a)\tilde{\mathbf{D}}-\tilde{\mathbf{D}}(\tilde{\mathbf{F}}^a\tilde{\mathbf{D}}+\tilde{\mathbf{D}}\tilde{\mathbf{F}}^a-\tilde{\mathbf{F}}^a)+\tilde{\boldsymbol{\zeta}}_a=\mathbf{0}, \qquad \text{(S25)}
\end{aligned}
$$

which proves Equation (S14).

Last but not least, it is straightforward to show that the general solution of perturbation-strength derivatives of the density matrix $\mathbf{D}_\omega^{b_1\cdots b_n}$ takes the following form[1]

$$
\begin{aligned}
\mathbf{D}_\omega^{b_1\cdots b_n} &= \mathbf{D}_{\mathrm{P}}^{b_1\cdots b_n}+\mathbf{D}_{\mathrm{H}}^{b_1\cdots b_n}, \qquad \text{(S26)} \\
\mathbf{D}_{\mathrm{P}}^{b_1\cdots b_n} &= -\mathbf{K}_\omega^{(n-1)}+\mathbf{K}_\omega^{(n-1)}\mathbf{D}+\mathbf{D}\mathbf{K}_\omega^{(n-1)}, \qquad \text{(S27)} \\
\mathbf{D}_{\mathrm{H}}^{b_1\cdots b_n} &= \mathbf{D}\mathbf{X}_\omega^{b_1\cdots b_n}-\mathbf{X}_\omega^{b_1\cdots b_n}\mathbf{D}, \qquad \text{(S28)}
\end{aligned}
$$

where $\mathbf{K}_{\omega}^{(n-1)}$ and $\mathbf{X}_{\omega}^{b_1\cdots b_n}$ are respectively

$$\begin{aligned}
\mathbf{K}_{\omega}^{(n-1)} &= \mathbf{Z}_{\omega}^{b_1\cdots b_n}\Big|_{\text{Remove terms involving } \mathbf{D}_{\omega}^{b_1\cdots b_n}} \\
&= \left[(\mathbf{DD})_{\omega}^{b_1\cdots b_n}\right]_{\text{Remove terms involving } \mathbf{D}_{\omega}^{b_1\cdots b_n}} \\
&= \sum_{P\subsetneq\{1,\cdots,n\}} \mathbf{D}_{\omega}^{b_P}\mathbf{D}_{\omega}^{b_{\{1,\cdots,n\}-P}}, \qquad \text{(S29)}
\end{aligned}$$

$$\left(\mathbf{E}^{[2]} - \omega_{B_N}\mathbf{S}^{[2]}\right)\mathbf{X}_{\omega}^{b_1\cdots b_n} = \mathbf{M}_{\omega}^{b_1\cdots b_n}, \qquad \text{(S30)}$$

with

$$\begin{aligned}
\mathbf{E}^{[2]}\mathbf{X}_{\omega}^{b_1\cdots b_n} &= \mathbf{G}^{\mathrm{KS}}([\mathbf{X}_{\omega}^{b_1\cdots b_n}, \mathbf{D}])\mathbf{D} - \mathbf{D}\mathbf{G}^{\mathrm{KS}}([\mathbf{X}_{\omega}^{b_1\cdots b_n}, \mathbf{D}]) \\
&\quad + \mathbf{F}[\mathbf{X}_{\omega}^{b_1\cdots b_n}, \mathbf{D}] - [\mathbf{X}_{\omega}^{b_1\cdots b_n}, \mathbf{D}]\mathbf{F}, \qquad \text{(S31)} \\
\mathbf{S}^{[2]}\mathbf{X}_{\omega}^{b_1\cdots b_n} &= [\mathbf{X}_{\omega}^{b_1\cdots b_n}, \mathbf{D}], \qquad \text{(S32)} \\
\mathbf{M}_{\omega}^{b_1\cdots b_n} &= \mathbf{Y}_{\omega}^{b_1\cdots b_n}\Big|_{\mathbf{D}_{\omega}^{b_1\cdots b_n}\to\mathbf{D}_{\mathrm{P}}^{b_1\cdots b_n}} \\
&= \left[(\mathbf{FD})_{\omega}^{b_1\cdots b_n} - (\mathbf{DF})_{\omega}^{b_1\cdots b_n} - \dot{\mathbf{D}}_{\omega}^{b_1\cdots b_n}\right]_{\mathbf{D}_{\omega}^{b_1\cdots b_n}\to\mathbf{D}_{\mathrm{P}}^{b_1\cdots b_n}}, \qquad \text{(S33)}
\end{aligned}$$

and $\mathbf{G}^{\mathrm{KS}}([\mathbf{X}_{\omega}^{b_1\cdots b_n}, \mathbf{D}])$ defined in Equation (163) of Reference 1.

References

(1) Thorvaldsen, A. J.; Ruud, K.; Kristensen, K.; Jørgensen, P.; Coriani, S. A Density Matrix-Based Quasienergy Formulation of the Kohn–Sham Density Functional Response Theory Using Perturbation- and Time-Dependent Basis Sets. *J. Chem. Phys.* **2008**, *129*, 214108.
