# Supplementary material for: Unified Framework for Molecular Response Functions of Different Electronic-Structure Models
Source: J Phys Chem A. 2025 Apr 16;129(16):3709–21. doi: 10.1021/acs.jpca.4c07789 (PMC12035861; doi:10.1021/acs.jpca.4c07789)
Supplement: Supplementary file 3 — jp4c07789_si_003.pdf [file jp4c07789_si_003.pdf]

Unified Framework for Molecular Response Functions of Different Electronic-Structure Models

Bin Gao* and Magnus Ringholm

Hylleraas Centre for Quantum Molecular Sciences, Department of Chemistry, UiT The Arctic University of Norway, N–9037 Tromsø, Norway

E-mail: bin.gao@uit.no

Response functions `La_bc_3` and `La_bc_2` computed in Listing 1 can be converted into LaTeX by simply calling the function `latexify` from the library Tinned,[1] as shown in Listing S1.

```
#include <iostream>
std::cout << "L^{abc}_{k_{\\rho}=3} = " << Tinned::latexify(La_bc_3) << "\n";
std::cout << "L^{abc}_{k_{\\rho}=2} = " << Tinned::latexify(La_bc_2) << "\n";
```

Listing S1: Snippet for converting response functions of the two-level atom into LaTeX.

The converted LaTeX can be compiled into the following expressions:

$$L^{abc}_{k_\rho=3} = \mathrm{tr}(\boldsymbol{\rho}^{c,b}\boldsymbol{V}^{a}_{\boldsymbol{\alpha}}), \tag{S1}$$

$$\begin{aligned} L^{abc}_{k_\rho=2} &= -\mathrm{tr}((\boldsymbol{\rho}^{c}\boldsymbol{\rho}^{b}+\boldsymbol{\rho}^{b}\boldsymbol{\rho}^{c})(\boldsymbol{V}^{a}_{\boldsymbol{\alpha}}\boldsymbol{\rho}+\boldsymbol{\rho}\boldsymbol{V}^{a}_{\boldsymbol{\alpha}}-\boldsymbol{V}^{a}_{\boldsymbol{\alpha}})) \\ &\quad -\mathrm{tr}((\boldsymbol{V}^{c}_{\boldsymbol{\gamma}}\boldsymbol{\rho}^{b}+\boldsymbol{V}^{b}_{\boldsymbol{\beta}}\boldsymbol{\rho}^{c}-\boldsymbol{\rho}^{c}\boldsymbol{V}^{b}_{\boldsymbol{\beta}}-\boldsymbol{\rho}^{b}\boldsymbol{V}^{c}_{\boldsymbol{\gamma}})(\boldsymbol{\rho}^{a}\boldsymbol{\rho}-\boldsymbol{\rho}\boldsymbol{\rho}^{a})), \end{aligned} \tag{S2}$$

and whose correctness can be easily verified.

Evaluation of response functions of the two-level atom requires the solution of the n-th order derivative of the density operator $\hat{\rho}_{\omega,jk}^{b_1\cdots b_n}$ $(j,k=0,1)$. The n-th order perturbed density operator can be obtained from the time integration of that at the $(n-1)$-th order[2]

$$\hat{\rho}_{jk}^{(n)}(t) = -\mathrm{i}\mathrm{e}^{-\mathrm{i}\omega_{jk}t}\int_{-\infty}^{t}[\hat{V}(\tau)\mathrm{e}^{\epsilon\tau}, \hat{\rho}^{(n-1)}(\tau)]_{jk}\mathrm{e}^{\mathrm{i}\omega_{jk}\tau}\mathrm{d}\tau, \tag{S3}$$

where the transition angular frequency $\omega_{jk} = E_j - E_k$.

Notice that the density operator $\hat{\rho}(t)$ can also be expanded in orders of perturbation strengths[3]

$$\begin{aligned}\hat{\rho}(t) &= \hat{\rho}^{(0)} + \hat{\rho}^{(1)}(t) + \cdots + \hat{\rho}^{(n-1)}(t) + \hat{\rho}^{(n)}(t) + \cdots \\ &= \hat{\rho}^{(0)} + \sum_{\omega_{B_1}}\mathrm{e}^{-\mathrm{i}\omega_{B_1}t}\varepsilon_{\omega_{B_1}}\hat{\rho}_{\omega}^{b_1} + \cdots \\ &+ \frac{1}{(n-1)!}\sum_{\omega_{B_1},\cdots,\omega_{B_{n-1}}}\mathrm{e}^{-\mathrm{i}\omega_{B_{N-1}}t}\varepsilon_{\omega_{B_1}}\cdots\varepsilon_{\omega_{B_{n-1}}}\hat{\rho}_{\omega}^{b_1\cdots b_{n-1}} \qquad \text{(S4)} \\ &+ \frac{1}{n!}\sum_{\omega_{B_1},\cdots,\omega_{B_n}}\mathrm{e}^{-\mathrm{i}\omega_{B_N}t}\varepsilon_{\omega_{B_1}}\cdots\varepsilon_{\omega_{B_n}}\hat{\rho}_{\omega}^{b_1\cdots b_n} + \cdots\,, \qquad \text{(S5)}\end{aligned}$$

we have

$$\begin{aligned}\hat{\rho}_{jk}^{(n)}(t) &\overset{(65)}{=} -\mathrm{i}\mathrm{e}^{-\mathrm{i}\omega_{jk}t}\int_{-\infty}^{t}[\sum_{\omega_{B_n}}\hat{B}_n\varepsilon_{\omega_{B_n}}\mathrm{e}^{-\mathrm{i}\omega_{B_n}\tau}\mathrm{e}^{\epsilon\tau}, \hat{\rho}^{(n-1)}(\tau)]_{jk}\mathrm{e}^{\mathrm{i}\omega_{jk}\tau}\mathrm{d}\tau \\ &\overset{(S4)}{=} -\frac{\mathrm{i}}{(n-1)!}\sum_{\omega_{B_1},\cdots,\omega_{B_n}}\mathrm{e}^{-\mathrm{i}\omega_{jk}t}\varepsilon_{\omega_{B_1}}\cdots\varepsilon_{\omega_{B_n}}\int_{-\infty}^{t}[\hat{B}_n, \hat{\rho}_{\omega}^{b_1\cdots b_{n-1}}]_{jk}\mathrm{e}^{\mathrm{i}(\omega_{jk}-\omega_{B_N})\tau+\epsilon\tau}\mathrm{d}\tau \\ &\overset{\epsilon\to 0}{=} -\frac{1}{(n-1)!}\sum_{\omega_{B_1},\cdots,\omega_{B_n}}\mathrm{e}^{-\mathrm{i}\omega_{B_N}t}\varepsilon_{\omega_{B_1}}\cdots\varepsilon_{\omega_{B_n}}\frac{[\hat{B}_n, \hat{\rho}_{\omega}^{b_1\cdots b_{n-1}}]_{jk}}{\omega_{jk}-\omega_{B_N}}, \qquad \text{(S6)}\end{aligned}$$

and the solution of the n-th order derivative of the density operator can be recognized as

$$\hat{\rho}_{\omega,jk}^{b_1\cdots b_n} = \sum_{m=1}^{n}\frac{[\hat{B}_m, \hat{\rho}_{\omega}^{b_{\{1,\cdots,n\}-\{m\}}}]_{jk}}{\omega_{B_1}+\cdots+\omega_{B_n}-\omega_{jk}}, \tag{S7}$$

by comparing to Equation (S5).

The evaluation of the derivatives of the density operator (S7) has been implemented into the library Tinned,[1] as well as other neccessary operators for the two-level atom. In Listing S2, we give a complete snippet for the evaluation of response functions La_bc_3 and La_bc_2 from Listing 1.

```
#include <map>
#include <symengine/constants.h>
#include <symengine/matrices/diagonal_matrix.h>
#include <symengine/matrices/immutable_dense_matrix.h>
#include <Tinned/TwoLevelAtom.hpp>
auto val_H0 = SymEngine::diagonal_matrix({
    SymEngine::symbol("E_0"), SymEngine::symbol("E_1")
});
auto val_Ba = SymEngine::immutable_dense_matrix(
    2, 2,
    {
        SymEngine::symbol("V_{\\alpha,00}"),
        SymEngine::symbol("V_{\\alpha,01}"),
        SymEngine::symbol("V_{\\alpha,10}"),
        SymEngine::symbol("V_{\\alpha,11}")
    }
);
auto val_Bb = SymEngine::immutable_dense_matrix(
    2, 2,
    {
        SymEngine::symbol("V_{\\beta,00}"),
        SymEngine::symbol("V_{\\beta,01}"),
        SymEngine::symbol("V_{\\beta,10}"),
        SymEngine::symbol("V_{\\beta,11}")
    }
);
auto val_Bc = SymEngine::immutable_dense_matrix(
```

```
    2, 2,
    {
        SymEngine::symbol("V_{\\gamma,00}"),
        SymEngine::symbol("V_{\\gamma,01}"),
        SymEngine::symbol("V_{\\gamma,10}"),
        SymEngine::symbol("V_{\\gamma,11}")
    }
);
auto val_rho0 = SymEngine::diagonal_matrix({SymEngine::one, SymEngine::zero});
auto fun_eval = Tinned::TwoLevelFunction(
    std::make_pair(H0, val_H0),
    std::map<SymEngine::RCP<const Tinned::OneElecOperator>,
             SymEngine::RCP<const SymEngine::MatrixExpr>,
             SymEngine::RCPBasicKeyLess>({
                 {Va, val_Ba}, {Vb, val_Bb}, {Vc, val_Bc}
             }),
    std::make_pair(rho, val_rho0)
);
auto val_La_bc_3 = fun_eval.apply(La_bc_3);
auto val_La_bc_2 = fun_eval.apply(La_bc_2);
```

Listing S2: Snippet for evaluating response functions (S1) and (S2) of the two-level atom.

The evaluated response functions `val_La_bc_3` and `val_La_bc_2` can also be converted

into LaTeX by using the function `latexify`. The following two expressions

$$L^{abc}_{k_\rho=3} = \frac{(-\frac{V_{\beta,01}V_{\gamma,00}}{(-(E_0-E_1)+\omega_\beta)} + \frac{V_{\beta,01}V_{\gamma,11}}{(-(E_0-E_1)+\omega_\beta)} + \frac{V_{\beta,11}V_{\gamma,01}}{(-(E_0-E_1)+\omega_\gamma)} - \frac{V_{\beta,00}V_{\gamma,01}}{(-(E_0-E_1)+\omega_\gamma)})V_{\alpha,10}}{(-(E_0-E_1)+\omega_\gamma+\omega_\beta)}$$
$$+ \frac{(-\frac{V_{\beta,10}V_{\gamma,01}}{(-(-E_0+E_1)+\omega_\beta)} - \frac{V_{\beta,01}V_{\gamma,10}}{(-(E_0-E_1)+\omega_\beta)} - \frac{V_{\beta,10}V_{\gamma,01}}{(-(E_0-E_1)+\omega_\gamma)} - \frac{V_{\beta,01}V_{\gamma,10}}{(-(-E_0+E_1)+\omega_\gamma)})V_{\alpha,11}}{(\omega_\gamma+\omega_\beta)}$$
$$+ \frac{(\frac{V_{\beta,10}V_{\gamma,01}}{(-(-E_0+E_1)+\omega_\beta)} + \frac{V_{\beta,01}V_{\gamma,10}}{(-(E_0-E_1)+\omega_\beta)} + \frac{V_{\beta,10}V_{\gamma,01}}{(-(E_0-E_1)+\omega_\gamma)} + \frac{V_{\beta,01}V_{\gamma,10}}{(-(-E_0+E_1)+\omega_\gamma)})V_{\alpha,00}}{(\omega_\gamma+\omega_\beta)}$$
$$+ \frac{(\frac{V_{\beta,10}V_{\gamma,11}}{(-(-E_0+E_1)+\omega_\beta)} - \frac{V_{\beta,10}V_{\gamma,00}}{(-(-E_0+E_1)+\omega_\beta)} + \frac{V_{\beta,11}V_{\gamma,10}}{(-(-E_0+E_1)+\omega_\gamma)} - \frac{V_{\beta,00}V_{\gamma,10}}{(-(-E_0+E_1)+\omega_\gamma)})V_{\alpha,01}}{(-(-E_0+E_1)+\omega_\gamma+\omega_\beta)}, \quad \text{(S8)}$$

and

$$L^{abc}_{k_\rho=2} = -((-\frac{V_{\beta,01}V_{\gamma,10}}{(-(-E_0+E_1)+\omega_\gamma)(-(E_0-E_1)+\omega_\beta)} - \frac{V_{\beta,10}V_{\gamma,01}}{(-(E_0-E_1)+\omega_\gamma)(-(-E_0+E_1)+\omega_\beta)})V_{\alpha,00}$$
$$-(-\frac{V_{\beta,01}V_{\gamma,10}}{(-(-E_0+E_1)+\omega_\gamma)(-(E_0-E_1)+\omega_\beta)} - \frac{V_{\beta,10}V_{\gamma,01}}{(-(E_0-E_1)+\omega_\gamma)(-(-E_0+E_1)+\omega_\beta)})V_{\alpha,11})$$
$$-(\frac{(\frac{V_{\beta,10}V_{\gamma,11}}{(-(-E_0+E_1)+\omega_\beta)} - \frac{V_{\beta,10}V_{\gamma,00}}{(-(-E_0+E_1)+\omega_\beta)} + \frac{V_{\beta,11}V_{\gamma,10}}{(-(-E_0+E_1)+\omega_\gamma)} - \frac{V_{\beta,00}V_{\gamma,10}}{(-(-E_0+E_1)+\omega_\gamma)})V_{\alpha,01}}{(-(E_0-E_1)+\omega_\alpha)}$$
$$+ \frac{(-\frac{V_{\beta,01}V_{\gamma,00}}{(-(E_0-E_1)+\omega_\beta)} + \frac{V_{\beta,01}V_{\gamma,11}}{(-(E_0-E_1)+\omega_\beta)} + \frac{V_{\beta,11}V_{\gamma,01}}{(-(E_0-E_1)+\omega_\gamma)} - \frac{V_{\beta,00}V_{\gamma,01}}{(-(E_0-E_1)+\omega_\gamma)})V_{\alpha,10}}{(-(-E_0+E_1)+\omega_\alpha)}), \quad \text{(S9)}$$

are compiled from the outcome of the function `latexify`. They are actually equivalent by recalling that $\omega_\alpha = -\omega_\beta - \omega_\gamma$.

References

(1) Gao, B. Tinned. 2024; `https://github.com/bingao/tinned`, a set of nonnumerical routines for computational chemistry.

(2) Norman, P.; Ruud, K.; Saue, T. *Principles and Practices of Molecular Properties*; John Wiley & Sons, Ltd, 2018.

(3) Thorvaldsen, A. J.; Ruud, K.; Kristensen, K.; Jørgensen, P.; Coriani, S. A Density

Matrix-Based Quasienergy Formulation of the Kohn–Sham Density Functional Response Theory Using Perturbation- and Time-Dependent Basis Sets. *J. Chem. Phys.* **2008**, *129*, 214108.
