# Supplementary material for: Unified Framework for Molecular Response Functions of Different Electronic-Structure Models
Source: J Phys Chem A. 2025 Apr 16;129(16):3709–21. doi: 10.1021/acs.jpca.4c07789 (PMC12035861; doi:10.1021/acs.jpca.4c07789)
Supplement: Supplementary file 4 — jp4c07789_si_004.pdf [file jp4c07789_si_004.pdf]

Unified Framework for Molecular Response Functions of Different Electronic-Structure Models

Bin Gao* and Magnus Ringholm

Hylleraas Centre for Quantum Molecular Sciences, Department of Chemistry, UiT The Arctic University of Norway, N–9037 Tromsø, Norway

E-mail: bin.gao@uit.no

Response functions `L_ab` and `L_abc` computed in Listing 3 can be easily converted into LaTeX and compiled into the following symbolic expressions:

$$\begin{aligned}\langle\langle A;B\rangle\rangle_{\omega_B} \overset{\mathrm{Re}}{=} & \boldsymbol{\lambda}\boldsymbol{\tau}^{\dagger}\mathrm{e}^{\mathrm{ad}_{-(\boldsymbol{t}\boldsymbol{\tau}^{\mathrm{T}})}}((\mathrm{ad}_{\boldsymbol{t}^{b}\boldsymbol{\tau}^{\mathrm{T}}})(\mathrm{ad}_{\boldsymbol{t}^{a}\boldsymbol{\tau}^{\mathrm{T}}})(\boldsymbol{H_0})) - \mathrm{e}^{\mathrm{ad}_{-(\boldsymbol{t}\boldsymbol{\tau}^{\mathrm{T}})}}((\mathrm{ad}_{\boldsymbol{t}^{b}\boldsymbol{\tau}^{\mathrm{T}}})(\boldsymbol{V}_{\boldsymbol{A}}^{a}))\\ & - \mathrm{e}^{\mathrm{ad}_{-(\boldsymbol{t}\boldsymbol{\tau}^{\mathrm{T}})}}((\mathrm{ad}_{\boldsymbol{t}^{a}\boldsymbol{\tau}^{\mathrm{T}}})(\boldsymbol{V}_{\boldsymbol{B}}^{b})) - \boldsymbol{\lambda}\boldsymbol{\tau}^{\dagger}\mathrm{e}^{\mathrm{ad}_{-(\boldsymbol{t}\boldsymbol{\tau}^{\mathrm{T}})}}((\mathrm{ad}_{\boldsymbol{t}^{b}\boldsymbol{\tau}^{\mathrm{T}}})(\boldsymbol{V}_{\boldsymbol{A}}^{a}))\\ & - \boldsymbol{\lambda}\boldsymbol{\tau}^{\dagger}\mathrm{e}^{\mathrm{ad}_{-(\boldsymbol{t}\boldsymbol{\tau}^{\mathrm{T}})}}((\mathrm{ad}_{\boldsymbol{t}^{a}\boldsymbol{\tau}^{\mathrm{T}}})(\boldsymbol{V}_{\boldsymbol{B}}^{b})) + \mathrm{e}^{\mathrm{ad}_{-(\boldsymbol{t}\boldsymbol{\tau}^{\mathrm{T}})}}((\mathrm{ad}_{\boldsymbol{t}^{b}\boldsymbol{\tau}^{\mathrm{T}}})(\mathrm{ad}_{\boldsymbol{t}^{a}\boldsymbol{\tau}^{\mathrm{T}}})(\boldsymbol{H_0})),\end{aligned} \tag{S1}$$

and

$$
\begin{aligned}
\langle\langle A; B, C\rangle\rangle_{\omega_B,\omega_C} \overset{\text{Re}}{=} & \,\mathrm{e}^{\mathrm{ad}_{-(\boldsymbol{t}\boldsymbol{\tau}^{\mathrm{T}})}}((\mathrm{ad}_{\boldsymbol{t}^b\boldsymbol{\tau}^{\mathrm{T}}})(\mathrm{ad}_{\boldsymbol{t}^a\boldsymbol{\tau}^{\mathrm{T}}})(\boldsymbol{V}_{\boldsymbol{C}}^c)) + \mathrm{e}^{\mathrm{ad}_{-(\boldsymbol{t}\boldsymbol{\tau}^{\mathrm{T}})}}((\mathrm{ad}_{\boldsymbol{t}^c\boldsymbol{\tau}^{\mathrm{T}}})(\mathrm{ad}_{\boldsymbol{t}^b\boldsymbol{\tau}^{\mathrm{T}}})(\boldsymbol{V}_{\boldsymbol{A}}^a)) \\
& + \mathrm{e}^{\mathrm{ad}_{-(\boldsymbol{t}\boldsymbol{\tau}^{\mathrm{T}})}}((\mathrm{ad}_{\boldsymbol{t}^c\boldsymbol{\tau}^{\mathrm{T}}})(\mathrm{ad}_{\boldsymbol{t}^a\boldsymbol{\tau}^{\mathrm{T}}})(\boldsymbol{V}_{\boldsymbol{B}}^b)) + (-\boldsymbol{\lambda}^a\boldsymbol{\tau}^\dagger\mathrm{e}^{\mathrm{ad}_{-(\boldsymbol{t}\boldsymbol{\tau}^{\mathrm{T}})}}((\mathrm{ad}_{\boldsymbol{t}^c\boldsymbol{\tau}^{\mathrm{T}}})(\boldsymbol{V}_{\boldsymbol{B}}^b)) \\
& - \boldsymbol{\lambda}^c\boldsymbol{\tau}^\dagger\mathrm{e}^{\mathrm{ad}_{-(\boldsymbol{t}\boldsymbol{\tau}^{\mathrm{T}})}}((\mathrm{ad}_{\boldsymbol{t}^a\boldsymbol{\tau}^{\mathrm{T}}})(\boldsymbol{V}_{\boldsymbol{B}}^b)) + \boldsymbol{\lambda}\boldsymbol{\tau}^\dagger\mathrm{e}^{\mathrm{ad}_{-(\boldsymbol{t}\boldsymbol{\tau}^{\mathrm{T}})}}((\mathrm{ad}_{\boldsymbol{t}^c\boldsymbol{\tau}^{\mathrm{T}}})(\mathrm{ad}_{\boldsymbol{t}^a\boldsymbol{\tau}^{\mathrm{T}}})(\boldsymbol{V}_{\boldsymbol{B}}^b))) \\
& + (-\boldsymbol{\lambda}^a\boldsymbol{\tau}^\dagger\mathrm{e}^{\mathrm{ad}_{-(\boldsymbol{t}\boldsymbol{\tau}^{\mathrm{T}})}}((\mathrm{ad}_{\boldsymbol{t}^b\boldsymbol{\tau}^{\mathrm{T}}})(\boldsymbol{V}_{\boldsymbol{C}}^c)) - \boldsymbol{\lambda}^b\boldsymbol{\tau}^\dagger\mathrm{e}^{\mathrm{ad}_{-(\boldsymbol{t}\boldsymbol{\tau}^{\mathrm{T}})}}((\mathrm{ad}_{\boldsymbol{t}^a\boldsymbol{\tau}^{\mathrm{T}}})(\boldsymbol{V}_{\boldsymbol{C}}^c)) \\
& + \boldsymbol{\lambda}\boldsymbol{\tau}^\dagger\mathrm{e}^{\mathrm{ad}_{-(\boldsymbol{t}\boldsymbol{\tau}^{\mathrm{T}})}}((\mathrm{ad}_{\boldsymbol{t}^b\boldsymbol{\tau}^{\mathrm{T}}})(\mathrm{ad}_{\boldsymbol{t}^a\boldsymbol{\tau}^{\mathrm{T}}})(\boldsymbol{V}_{\boldsymbol{C}}^c))) + (-\boldsymbol{\lambda}^b\boldsymbol{\tau}^\dagger\mathrm{e}^{\mathrm{ad}_{-(\boldsymbol{t}\boldsymbol{\tau}^{\mathrm{T}})}}((\mathrm{ad}_{\boldsymbol{t}^c\boldsymbol{\tau}^{\mathrm{T}}})(\boldsymbol{V}_{\boldsymbol{A}}^a)) \\
& - \boldsymbol{\lambda}^c\boldsymbol{\tau}^\dagger\mathrm{e}^{\mathrm{ad}_{-(\boldsymbol{t}\boldsymbol{\tau}^{\mathrm{T}})}}((\mathrm{ad}_{\boldsymbol{t}^b\boldsymbol{\tau}^{\mathrm{T}}})(\boldsymbol{V}_{\boldsymbol{A}}^a)) + \boldsymbol{\lambda}\boldsymbol{\tau}^\dagger\mathrm{e}^{\mathrm{ad}_{-(\boldsymbol{t}\boldsymbol{\tau}^{\mathrm{T}})}}((\mathrm{ad}_{\boldsymbol{t}^c\boldsymbol{\tau}^{\mathrm{T}}})(\mathrm{ad}_{\boldsymbol{t}^b\boldsymbol{\tau}^{\mathrm{T}}})(\boldsymbol{V}_{\boldsymbol{A}}^a))) \\
& - \mathrm{e}^{\mathrm{ad}_{-(\boldsymbol{t}\boldsymbol{\tau}^{\mathrm{T}})}}((\mathrm{ad}_{\boldsymbol{t}^c\boldsymbol{\tau}^{\mathrm{T}}})(\mathrm{ad}_{\boldsymbol{t}^b\boldsymbol{\tau}^{\mathrm{T}}})(\mathrm{ad}_{\boldsymbol{t}^a\boldsymbol{\tau}^{\mathrm{T}}})(\boldsymbol{H}_{\boldsymbol{0}})) + (\boldsymbol{\lambda}^a\boldsymbol{\tau}^\dagger\mathrm{e}^{\mathrm{ad}_{-(\boldsymbol{t}\boldsymbol{\tau}^{\mathrm{T}})}}((\mathrm{ad}_{\boldsymbol{t}^c\boldsymbol{\tau}^{\mathrm{T}}})(\mathrm{ad}_{\boldsymbol{t}^b\boldsymbol{\tau}^{\mathrm{T}}})(\boldsymbol{H}_{\boldsymbol{0}})) \\
& + \boldsymbol{\lambda}^b\boldsymbol{\tau}^\dagger\mathrm{e}^{\mathrm{ad}_{-(\boldsymbol{t}\boldsymbol{\tau}^{\mathrm{T}})}}((\mathrm{ad}_{\boldsymbol{t}^c\boldsymbol{\tau}^{\mathrm{T}}})(\mathrm{ad}_{\boldsymbol{t}^a\boldsymbol{\tau}^{\mathrm{T}}})(\boldsymbol{H}_{\boldsymbol{0}})) + \boldsymbol{\lambda}^c\boldsymbol{\tau}^\dagger\mathrm{e}^{\mathrm{ad}_{-(\boldsymbol{t}\boldsymbol{\tau}^{\mathrm{T}})}}((\mathrm{ad}_{\boldsymbol{t}^b\boldsymbol{\tau}^{\mathrm{T}}})(\mathrm{ad}_{\boldsymbol{t}^a\boldsymbol{\tau}^{\mathrm{T}}})(\boldsymbol{H}_{\boldsymbol{0}})) \\
& - \boldsymbol{\lambda}\boldsymbol{\tau}^\dagger\mathrm{e}^{\mathrm{ad}_{-(\boldsymbol{t}\boldsymbol{\tau}^{\mathrm{T}})}}((\mathrm{ad}_{\boldsymbol{t}^c\boldsymbol{\tau}^{\mathrm{T}}})(\mathrm{ad}_{\boldsymbol{t}^b\boldsymbol{\tau}^{\mathrm{T}}})(\mathrm{ad}_{\boldsymbol{t}^a\boldsymbol{\tau}^{\mathrm{T}}})(\boldsymbol{H}_{\boldsymbol{0}}))), \qquad \text{(S2)}
\end{aligned}
$$

by using the function `latexify` from the library Tinned.[1] But one should note that each right-hand side term of Equations (S1) and (S2) is an expectation value. The notation incompleteness is due to the fact that we treat the exponential map as "an expectation value" in the implementation so that we avoid introducing an additional class only for expectation values in the library Tinned.

Right-hand sides (RHS) of response equations of coupled-cluster amplitudes and Lagrangian multipliers can also be computed in a symbolic manner by using the library SymResponse.[2] The following expressions are the RHSes of response equations of $\boldsymbol{t}_\omega^a$ and $\boldsymbol{\lambda}_\omega^a$, respectively

$$
-\boldsymbol{\xi}_\omega^a = -\mathrm{e}^{\mathrm{ad}_{-(\boldsymbol{t}\boldsymbol{\tau}^{\mathrm{T}})}}(\boldsymbol{V}_{\boldsymbol{A}}^a), \qquad \text{(S3)}
$$

$$
\begin{aligned}
-\boldsymbol{\zeta}_\omega^a = & \,\boldsymbol{\lambda}\boldsymbol{\tau}^\dagger\mathrm{e}^{\mathrm{ad}_{-(\boldsymbol{t}\boldsymbol{\tau}^{\mathrm{T}})}}((\mathrm{ad}_{\boldsymbol{\tau}})(\boldsymbol{V}_{\boldsymbol{A}}^a)) + \mathrm{e}^{\mathrm{ad}_{-(\boldsymbol{t}\boldsymbol{\tau}^{\mathrm{T}})}}((\mathrm{ad}_{\boldsymbol{\tau}})(\boldsymbol{V}_{\boldsymbol{A}}^a)) \\
& - \boldsymbol{\lambda}\boldsymbol{\tau}^\dagger\mathrm{e}^{\mathrm{ad}_{-(\boldsymbol{t}\boldsymbol{\tau}^{\mathrm{T}})}}((\mathrm{ad}_{\boldsymbol{\tau}})(\mathrm{ad}_{\boldsymbol{t}^a\boldsymbol{\tau}^{\mathrm{T}}})(\boldsymbol{H}_{\boldsymbol{0}})) - \mathrm{e}^{\mathrm{ad}_{-(\boldsymbol{t}\boldsymbol{\tau}^{\mathrm{T}})}}((\mathrm{ad}_{\boldsymbol{\tau}})(\mathrm{ad}_{\boldsymbol{t}^a\boldsymbol{\tau}^{\mathrm{T}}})(\boldsymbol{H}_{\boldsymbol{0}})), \qquad \text{(S4)}
\end{aligned}
$$

which can be evaluated and used to solve the corresponding response equations.

References

(1) Gao, B. Tinned. 2024; `https://github.com/bingao/tinned`, a set of nonnumerical routines for computational chemistry.

(2) Gao, B. SymResponse. 2024; `https://github.com/bingao/symresponse`, a unified framework for response theory at different levels of electronic-structure theory.
